# Structural Design of Poly(2-amino-2-oxazoline)s for Kinetic Hydrate Inhibition of Natural Gas and Methane Hydrates

**DOI:** 10.1021/acsomega.5c00143

**Published:** 2025-04-29

**Authors:** Malcolm A. Kelland, Somdeb Jana, Julie Kiær, Ajla Salihovic, Janronel Pomicpic, Richard Hoogenboom

**Affiliations:** † Department of Chemistry, Bioscience and Environmental Engineering, Faculty of Science and Technology, University of Stavanger, N-4036 Stavanger, Norway; ‡ Supramolecular Chemistry Group, Centre of Macromolecular Chemistry, Department of Organic and Macromolecular Chemistry, Ghent University, Krijgslaan 281—Building S4, B-9000 Ghent, Belgium

## Abstract

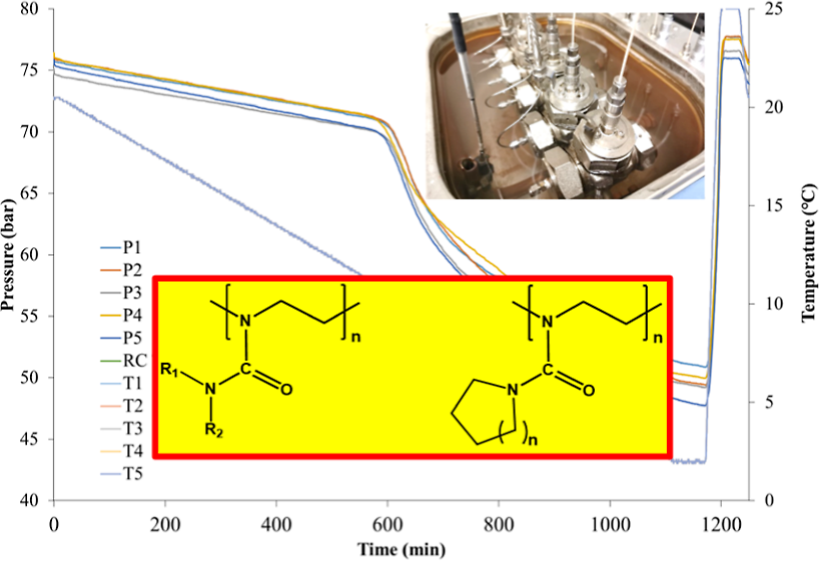

Kinetic hydrate inhibitors (KHIs) are chemical substances that prevent gas hydrate plugging of oil and gas production flow lines. The main ingredient in a KHI formulation is one or more water-soluble amphiphilic polymers. We recently presented the first results on the KHI performance of a new class of amphiphilic polymers, namely, poly(2-dialkylamino-2-oxazoline)s, which showed good potential as KHIs. In this work, this class of novel KHIs has been investigated in more detail using both structure I and structure II hydrate-forming gases to optimize the polymer structure for best performance and with higher cloud point temperature for wider field applications. All polymers were tested in high-pressure rocking cells using the slow (1 °C/h) constant cooling test method. The best poly(2-dialkylamino-2-oxazoline)s tested at 2500 ppm contained 5-membered and 6-membered heterocyclic pendant groups and performed similarly to a commercial KHI polymer, poly(*N*-vinyl caprolactam) (PVCap), with both gases and with higher cloud point temperature (*T*_CP_) than PVCap, thereby expanding the workability temperature range. The effect of salinity on KHI performance has also been studied, along with high flash point glycol solvents as synergists in combination with the best performing polymers. The onset temperature using the 2500–5000 ppm polymer could further be lowered by about 2–3 °C by the addition of 5000 ppm butylated glycol ethers. Hence, this work demonstrates the broader potential of poly(2-dialkylamino-2-oxazoline)s as KHIs.

## Introduction

Kinetic hydrate inhibitors (KHIs) are a class of low dosage hydrate inhibitors (LDHIs) that are based on one or more water-soluble polymers with added solvent and synergists.^1−10^ Their main application is to prevent unwanted gas hydrate formation and plugging in upstream multiphase well streams, either on land in cold climate or in subsea flow lines. KHIs are also applied in drilling and completion fluids, injection of CO_2_.^11−15^ KHIs are liquid formulations containing water-soluble polymers in various solvents. These solvents can be synergistic with the polymers, but other chemicals can also be added to boost the performance. The active polymer is present in about 5–25 wt % of the liquid formulation. A typical polymer dosage is about 1000–5000 ppm (0.1–0.5 wt %), meaning that KHI formulations are often injected as 0.5–5 wt % of the aqueous produced fluid.^16^

KHIs inhibit the hydrate formation process kinetically, either at the nucleation stage or additionally at the crystal growth stage. Operators want no macroscopic hydrate particles at all in their flow lines; therefore, complete hydrate inhibition by the KHI is the key target. One KHI polymer was reported to induce complete hydrate dissociation within the gas hydrate-stable pressure–temperature region.^17^ KHIs are limited in the application range by several factors, time and subcooling being the main parameters, but the system pressure is also an important factor. Subcooling (Δ*T*) is the temperature difference between the incumbent temperature and the equilibrium temperature at the operating pressure. Most KHI applications are at DT < 10–12 °C. One study suggested that the KHI could readily offer good protection for long periods of shut-in (e.g., >168 h at up to 15 °C subcooling) followed by restart.^18^

Currently, the water-soluble polymers used in commercial KHI formulations are polyamides in which the amide group is bonded to a hydrophobic group such as alkyl, cycloalkyl, or part of a lactam ring. Polyamine oxide groups, where the nitrogen atom is bonded to hydrophobic groups, also show good potential as KHI polymers, but they are currently not in commercial use.

It is our contention that a high-performing KHI polymer must have a hydrophobic group next to the amide, which should have an optimal size and shape, usually with 3–6 carbon atoms. These hydrophobic groups are presumed to interact with open cavities on the hydrate particles, imitating the alkane gas molecules that would otherwise be encapsulated to make clathrate gas hydrate cages. Polymers that fit these requirements are usually thermoresponsive with lower critical solution temperature behavior, i.e., they lose solubility in water above the *T*_CP_.

**Figure 1:**
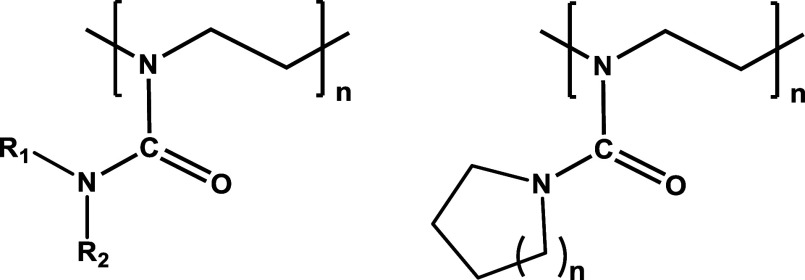
Structure of PAmOx polymers with acyclic or cyclic hydrophobic substituents.

Over the past decade, we have reported on a range of water-soluble polymer classes to demonstrate the aforementioned contention. Recently, we introduced a new class of polymers, poly(2-dialkylamino-2-oxazoline)s (PAmOx), as KHIs for a synthetic natural gas (SNG) mixture for the first time^19^ (Figure 1). SNG with water forms structure II clathrate hydrate as the most thermodynamically stable phase. These polymers comprise a tetraalkylurea structure in the repeat unit, in which one of the nitrogen atoms is part of the polymer backbone.^20^ These polymers are theoretically based on polymerization of 2-dialkyl-2-oxazoline (AmOx) monomers, which is why they are given the abbreviation PAmOx. These PAmOx polymers can also exhibit thermoresponsive behavior in water, depending on the size and shape of the side chain substituents.

Our initial KHI study was carried out using a natural gas blend that forms structure II clathrate hydrate as the most thermodynamically favored phase. The slow constant cooling method (1 °C/h) in multiple high-pressure steel rocking cells was used as the KHI ranking method. The onset temperature (*T*_o_), when the macroscopic hydrate was first observed from the pressure data, was used as the main parameter to gauge the KHI performance of the PAmOx polymers. The study determined that if the alkyl substituent was too small (e.g., methyl or ethyl), the KHI performance was poor. The best performance was found for 5–6-membered cyclic amine substituents (pyrrolidine and piperidine). However, the homopolymer based only on the piperidine monomer (PyOx) showed poor aqueous solubility, which affected the KHI performance negatively. Furthermore, if an oxygen atom was added to the ring to give a morpholine ring, the solubility increased, but the KHI performance was poor. These findings are in line with our general contention regarding the optimum parameters for the amphiphilic groups in KHI polymers.^21^ The best-performing PAmOx polymers also had low *T*_CP_ (down to 22 °C), which is not practical for injection into or use in hot aqueous fluids, especially saline fluids.

We hereby present an extended study on PAmOx polymers as KHIs, in which we have expanded the range of polymer structures (molar mass, copolymer ratio, and new monomers) to attempt to optimize the polymer performance while still keeping good water solubility. We have also investigated the performance of PAmOx polymers with methane as the gas phase, which forms structure I clathrate hydrate as the most thermodynamically favored phase. Finally, we investigate the KHI performance of some of the polymers in 3 wt % NaCl brine and with a range of high flash point glycol solvents, which have previously been shown to be good synergists for other polyamide KHI classes.

## Experimental Section

### Materials

**Figure 2:**
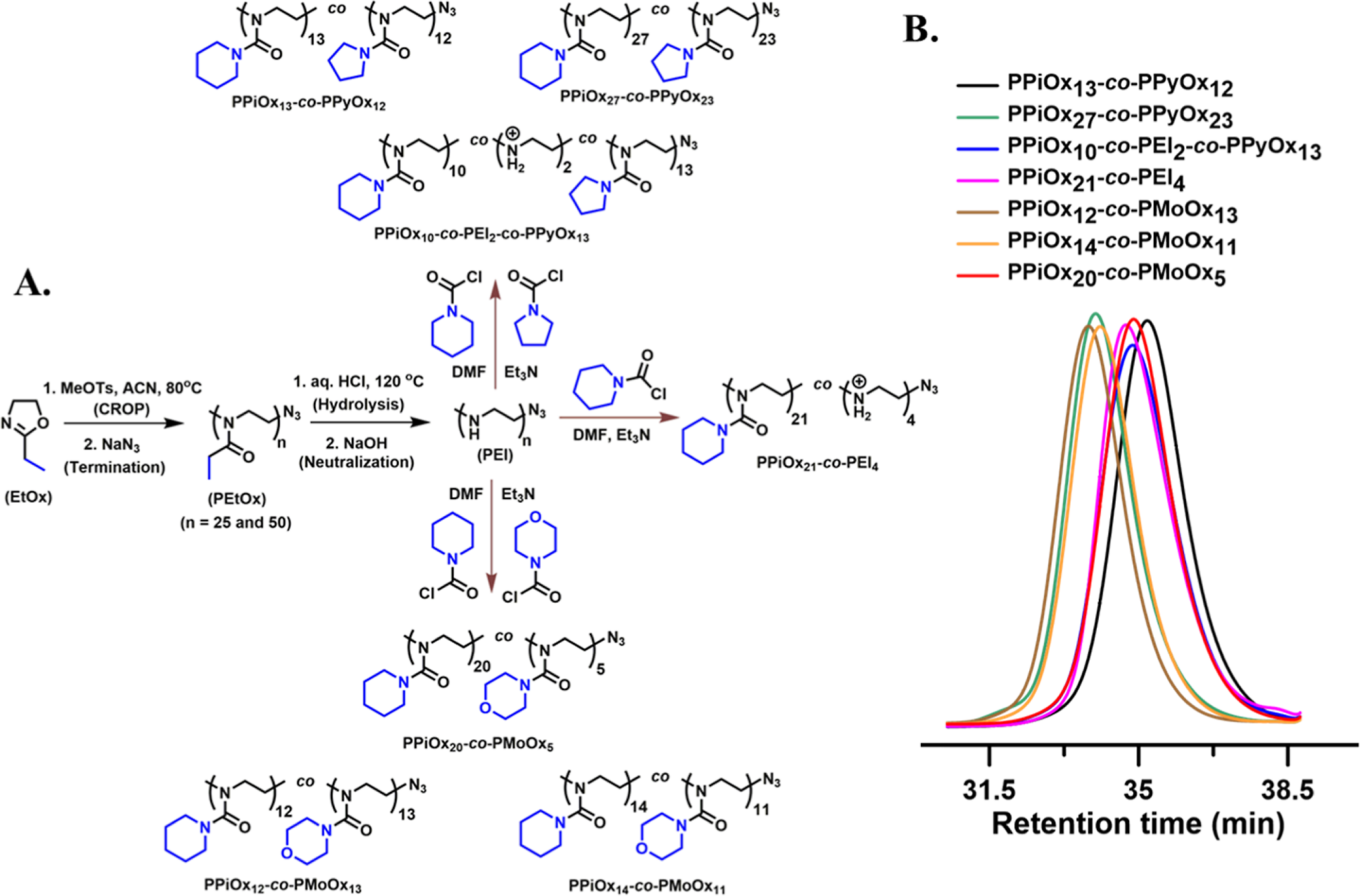
(A) Schematic illustration of the synthesis of different amine side chain functional poly(2-oxazoline) copolymers via acylation of linear PEI. (B) Normalized SEC traces of as-synthesized copolymers in DMA (in the presence of LiBr) eluent.

Poly(*N*-vinylcaprolactam) (PVCap) homopolymer (*M*_n_ = 2600 g/mol, 41.1 wt % in monoethylene glycol) was supplied by BASF. The solvent was removed before KHI testing by repeated precipitation above the solution *T*_CP_, followed by vacuum drying. The synthesis of different PAmOx via acylation of linear polyethylenimine (PEI) is summarized in Figure 2, and their characterization is summarized in Table 1. Table 1 also includes some polymers that were reported in our previous study (first three entries), two of which are also tested further in this study.^19^ Full details of the synthesis and characterization of all polymers can be found in the Supporting Information.

**Table 1:** Characteristics of the Synthesized Polymers

Polymer	*M*_n_ (theo) (kDa)	*M*_n_ (SEC) (kDa)	*D̵*	*T*_CP_ (°C)
PEtOx_25_	2.47	2.58	1.02	
PEtOx_50_	4.95	4.7	1.03	
PEI_25_	1.10			
PEI_50_	2.2			
PPyOx_25_	3.55	2.1	1.28	>70
PPiOx_25_	3.9	1.6	1.09	16^a^
PPiOx_14_-*co*-PPyOx_11_	3.7	2.0	1.18	26
PPiOx_27_-*co*-PPyOx_23_	7.4	3.2	1.17	28
PPiOx_13_-*co*-PPyOx_12_	3.7	1.97	1.18	36
PPiOx_10_-*co*-PEI_2_-*co*-PPyOx_13_	3.5	2.2	1.20	50
PPiOx_12_-*co*-PMoOx_13_	3.92	3.6	1.13	61
PPiOx_14_-*co*-PMoOx_11_	3.9	3.2	1.14	48
PPiOx_21_-*co*-PEI_4_	3.45	2.15	1.20	33
PPiOx_20_-*co*-PMoOx_5_	3.9	2.24	1.17	25

a Not completely soluble below the cloud point temperature (*T*_CP_).

### Kinetic Hydrate Inhibitor Performance Screening Tests

Test were carried out in a set of five parallel high-pressure rocking cells. The cells were rocked at 20 rocks/min and placed in a temperature-controlled water bath. The equipment including the software was supplied by PSL Systemtechnik, Germany, except the cells, which were made by Swagelok, Norway (Figure 4).^19,21^ Two gases were used for the KHI tests, a synthetic natural gas (SNG, Table 2) and methane (99.9%), both supplied by Yara Praxair, Norway. The SNG composition was analyzed to be within ±0.1% of all the required concentrations. The equilibrium temperatures (HETs) were determined using PVTSim software by Calsep, Denmark. HET was predicted to be 20.5 °C for the structure II gas hydrate with 76 bar of SNG and 15.7 °C for 110 bar methane.

**Figure 3:**
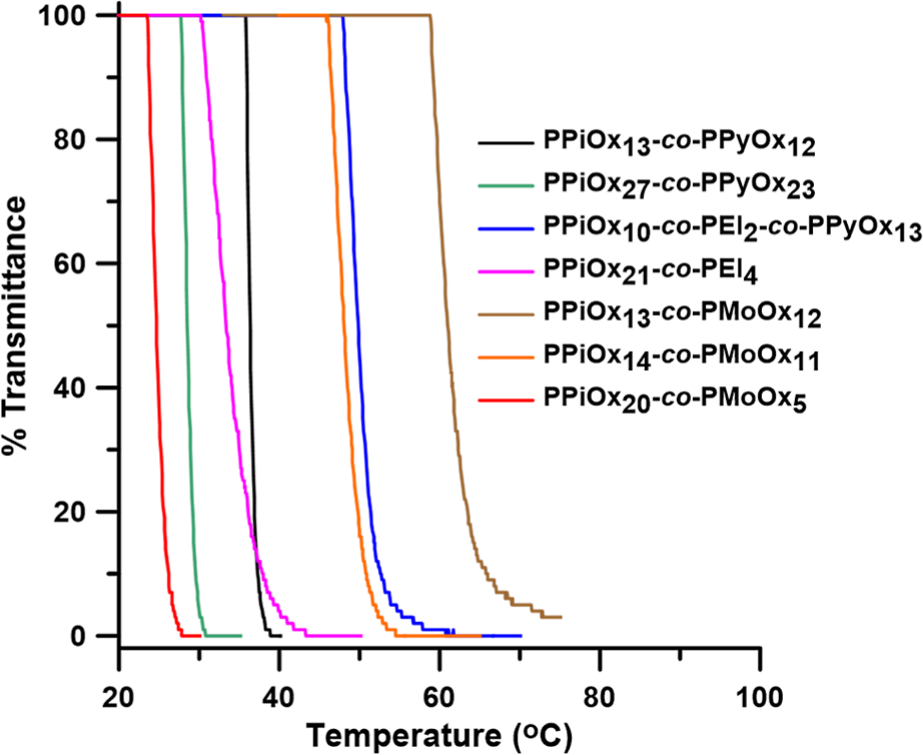
LCST-type turbidimetry (heating) curves of synthesized PAmOx copolymers in aqueous medium (sample conc. 2 mg/mL).

**Figure 4:**
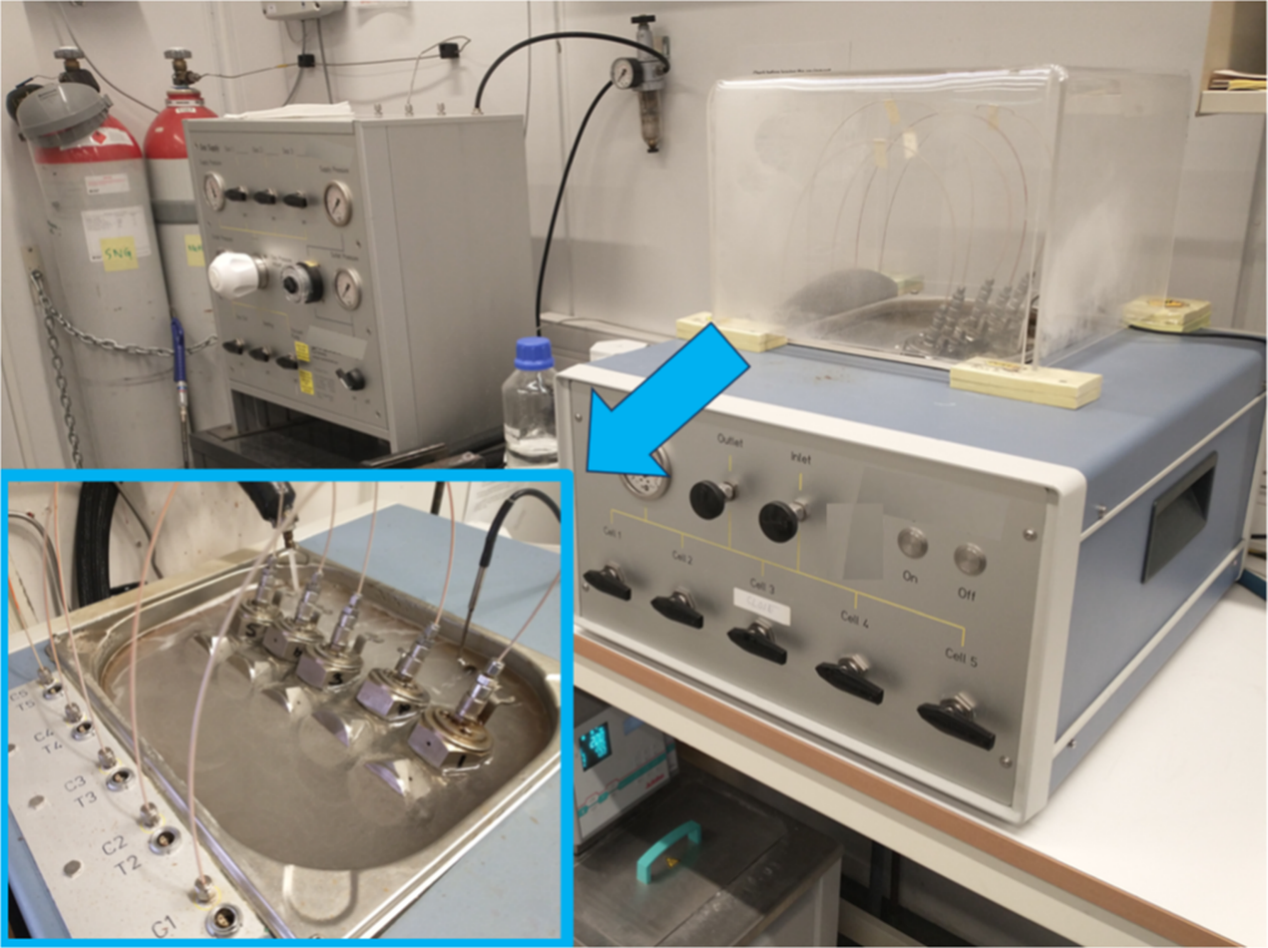
High-pressure steel multicell rocking in a temperature-controlled bath.

**Table 2:** Composition of the Synthetic Natural Gas Mixture

Component	mol %
Nitrogen	0.11
*n*-Butane	0.72
Isobutane	1.65
Propane	5.00
CO_2_	1.82
Ethane	10.3
Methane	80.4

The slow constant cooling (SCC) screening test method was used to evaluate the KHI performance of all polymers. This method has been used by us for over a decade in the same equipment and test conditions in order to be able to compare various classes of KHIs. This includes the same initial pressure, gas composition, cooling rate, and rocking rate. In this study, we used both 20 mL of aqueous fluid in each cell (the standard volume) and 10 mL. The lower amount of fluid was used due to the limited amount of each polymer available. The SCC test procedure was as follows:

1. The test polymer was dissolved in 105 mL of deionized water 1 day prior to testing. The selected volume of test solution (10 or 20 mL) was added to each cell.
2. All five cells were purged with SNG, and then, vacuum was applied to remove air. The process was then repeated.
3. The test gas was loaded to each cell at room temperature (20.5 °C) (76 bar SNG or 110 bar methane), and each cell was shut individually at the gas inlet/outlet valves.
4. The cells were rocked and slowly cooled at a rate of 1 °C/h, and the pressure and temperature data were recorded.

**Figure 5:**
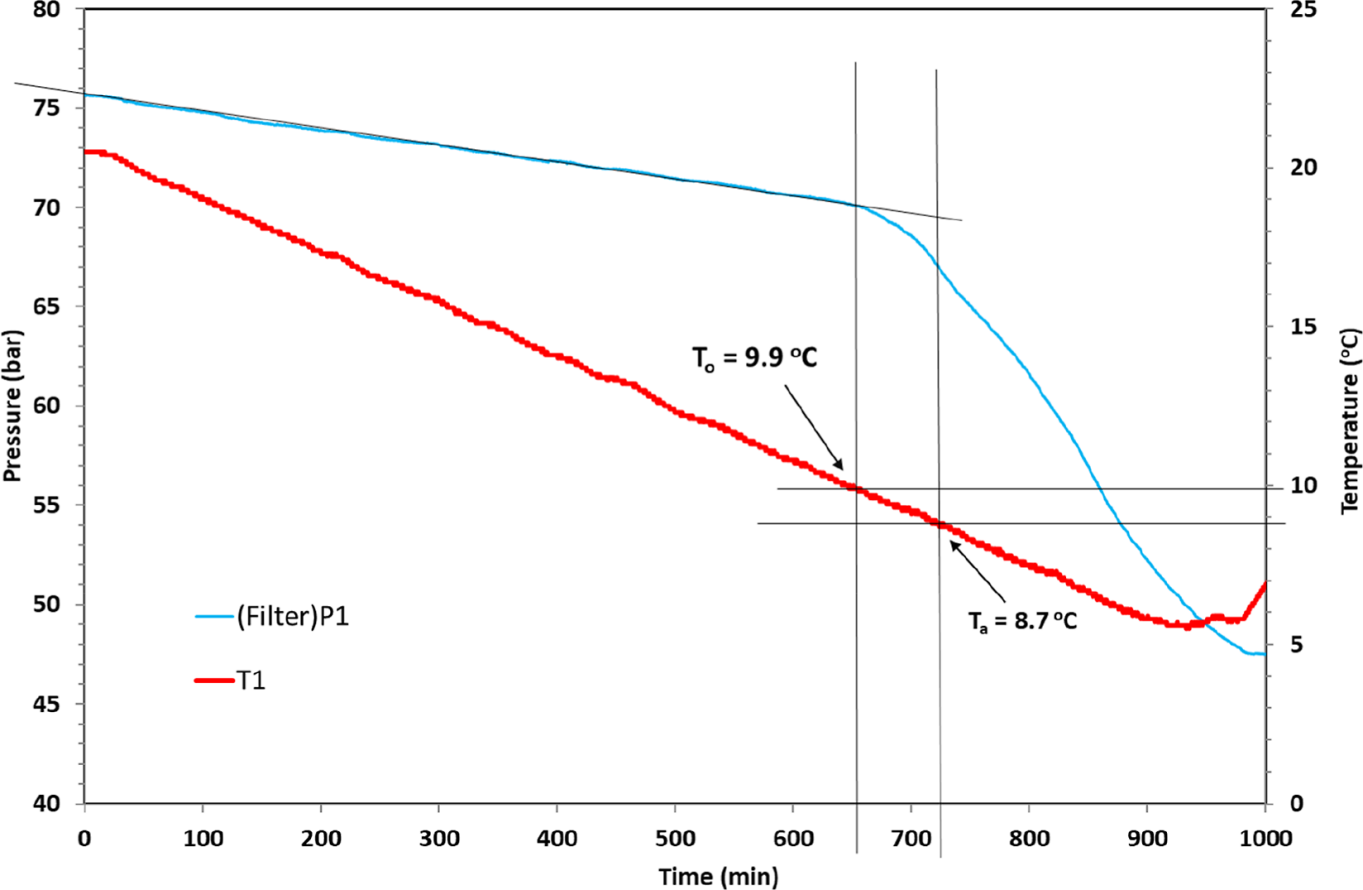
Example of PT data vs time graph for a SCC test with SNG and determination of *T*_o_ and *T*_a_ values.

From the rocking cell tests, two main parameters were derived, the hydrate onset temperature (*T*_o_) and the rapid hydrate formation temperature (*T*_a_) (Figure 5). As the cells are closed, the pressure decreases linearly as the temperature decreases constantly. At some point, gas hydrates start to form, and the pressure then deviates from the linear decreasing trend. The point of this first pressure drop is called *P*_o_, and the corresponding temperature at *P*_o_ is called *T*_o_. *T*_o_ is determined to be 9.9 °C in the graph in Figure 5. After *T*_o_, the pressure drops faster and faster until it reaches the highest pressure drop rate of the experiment. This point is called *P*_a_, and its corresponding temperature is *T*_a_. The value of *T*_a_ is 8.7 °C, as shown in Figure 5. For each polymer sample, 5–10 individual cell experiments were carried out. For such a set of experiments, we typically observe a 10–15% margin of error in *T*_o_ and *T*_a_ values. This was also the case in this study. A typical full set of data for 6 cells have been reported.^22^ The stochastic nature of the hydrate nucleation process is the main cause of the variation in the *T*_o_ and *T*_a_ values. No bias was observed between any of the cells, such as one cell regularly giving higher or lower *T*_o_ and *T*_a_ values than the others.

The *T*_o_ value is a more important parameter than *T*_a_ for determining the KHI performance because oil and gas companies want to ensure complete prevention of gas hydrates in their flow lines. To evaluate if there is a significant difference between sets of *T*_o_ values for two polymers, we carried out statistical *t* tests and determined the *p*-value. If the *p*-value is less than 0.05, we consider that there is a significant difference between the *T*_o_ values and thus the performance of the KHIs at the 95% confidence level.^23^

## Results and Discussion

### Polymer Synthesis

Very recently, we reported the first results on the KHI performance of a new class of amphiphilic polymers, namely, poly(2-amino-2-oxazoline)s (PAmOx), which showed promising potential as KHIs.^19^ As per our previous studies, the best PAmOx contained 6-membered piperidinyl and 5-membered pyrrolidinyl heterocyclic pendant groups and performed similar to a commercial KHI polymer, poly(*N*-vinyl caprolactam) (PVCap). Here, in this report, further attempts have been made to broaden the potential of poly(2-amino-2-oxazoline)s as KHIs by varying molecular weight, composition, and different other substituents. Newly synthesized KHIs have been investigated in more detail using both structure I and structure II hydrate-forming gases to optimize the polymer structure for best performance and with higher cloud point temperature (*T*_CP_) for wider field applications. The strategies for the synthesis of different PAmOx (co)polymers employed in this work are depicted in Figure 2A. The synthetic methodology is based on the acylation of the linear polyethylenimine (PEI) reported previously.^20,35,36^ The protocol is straightforward and allows us to synthesize well-defined functional PAmOx. It should be noted that the well-defined PAmOx are difficult to synthesize by cationic ring-opening polymerization (CROP) of corresponding 2-oxazoline monomers because of intrinsic chain transfer and chain termination side reactions.^37,38^ In contrast, acylation of PEI is well-suited for the quick synthesis of well-defined poly(2-oxazoline)s libraries with desired side-chain functional groups and thus minimizes tedious monomer synthesis and purification steps for each different poly(2-oxazoline)s. Herein, the starting materials, i.e., linear PEI with a degree of polymerization (DP) of 25 and 50, were synthesized via living CROP of 2-ethyl-2-oxazoline (EtOx) followed by the controlled acid-induced hydrolysis of the resulting poly(2-ethyl-2-oxazoline) (PEtOx; *M*_n_ = 2.58 and 4.7 kDa with a *D̵* of 1.02 and 1.03, respectively) (Figure 2A, Table 1). The degree of polymerization (DPn) of the synthesized PEtOx was also determined using ^1^H NMR spectroscopy by calculating the integration ratio of the methylene protons (at δ 3.41 ppm) in the polymer backbone to the ω-terminal methylene protons (δ 4.31 ppm) adjacent to the azide end group (Figure S1). The complete hydrolysis of PEtOx to PEI was confirmed by ^1^H NMR spectroscopy, where the proton signals (at δ 0.99 and 2.2 ppm) corresponding to the PEtOx side-chains (−CH_2_CH_3_) (Figure S1) are completely disappeared and the main backbone ethylene (−N–CH_2_CH_2_) signal is switched from δ 3.41 ppm (PEtOx ethylene protons adjacent to the amide) (Figure S1) to δ 2.75 ppm (PEI ethylene protons adjacent to the amine) (Figures S3 and S4). In the next step, amine side chain functional copolymers, namely, poly(2-piperidinyl-2-oxazoline)-*co*-poly(2-pyrrolidinyl-2-oxazoline) (PPiOx-*co*-PPyOx) with different molecular weights and compositions (such as PPiOx_27_-*co*-PPyOx_23_, PPiOx_13_-*co*-PPyOx_12_), were synthesized via the controlled one-pot reacylation of linear PEI in the presence of pyrrolidine carbonyl chloride and piperidinyl carbonyl chloride (Figure 2A). Poly(2-piperidinyl-2-oxazoline)-*co*-poly(2-morpholinyl-2-oxazoline) (PPiOx-*co*-PMoOx) with different compositions (such as PPiOx_12_-*co*-PMoOx_13_, PPiOx_14_-*co*-PMoOx_11_, and PPiOx_20_-*co*-PMoOx_5_) were also synthesized via a similar approach, as depicted in Figure 2A, to observe the impact of the hydrophilic six-membered morpholine moiety on the KHI efficiency. Additionally, PAmOx copolymers with cationic functional groups, such as PPiOx_21_-*co*-PEI_4_ and PPiOx_10_-*co*-PEI_2_-*co*-PPyOx_13_, were also synthesized via controlled and partial modification of PEI in the presence of piperidine carbonyl chloride and/or pyrrolidine carbonyl chloride (Figure 2A) to investigate their KHI performance. The presence of hydrophilic segments and cationic charge would also increase the cloud point of the copolymer compared to that of the PPiOx homopolymer (*T*_CP_ ∼ 16 °C), and a high cloud point is considered as beneficial for injecting KHIs into hot and/or high-salinity well streams to avoid KHI polymer precipitation.^16,21^ Our previous study has shown that piperidinyl is an active moiety for KHI, and therefore, newly synthesized PPiOx-based copolymers with varying substituents, composition, and molecular weight were anticipated to have a major influence on the KHI performance.

The newly synthesized amine side-chain functional nonionic as well as ionic copolymers were analyzed by means of ^1^H NMR spectroscopy, size exclusion chromatography (SEC), and turbidimetry (Figure 2B, ESI and Table 1). In the case of nonionic copolymers (i.e., PPiOx_27_-*co*-PPyOx_23_, PPiOx_13_-*co*-PPyOx_12_, PPiOx_12_-*co*-PMoOx_13_, PPiOx_14_-*co*-PMoOx_11_, and PPiOx_20_-*co*-PMoOx_5_), the full conversion of the PEI acylation was established by ^1^H NMR spectroscopy. The ^1^H NMR revealed the complete disappearance of the PEI signal at δ 2.75 ppm (Figures S3 and S4), while new peaks from the poly(2-oxazoline) backbone (δ 3.2–3.4 ppm) and amine side chains appeared (Figures S5, S6, S11, S12, and S13). The compositions of pyrrolidine, piperidine, and morpholine units in the PPiOx_27_-*co*-PPyOx_23_, PPiOx_13_-*co*-PPyOx_12_, PPiOx_12_-*co*-PMoOx_13_, PPiOx_14_-*co*-PMoOx_11_, and PPiOx_20_-*co*-PMoOx_5_ copolymers were determined from the integration of the appropriate signals in the ^1^H NMR spectrum of the respective samples (Figures S5, S6, S11, S12, and S13). It should be noted that the composition of PEI segments in the ionic PPiOx_21_-*co*-PEI_4_ and PPiOx_10_-*co*-PEI_2_-*co*-PPyOx_13_ copolymers cannot be determined directly from their corresponding ^1^H NMR spectrum because of the overlapping of characteristic signals (Figures S7 and S9). Therefore, a few milligrams of synthesized ionic copolymers (PPiOx_21_-*co*-PEI_4_ and PPiOx_10_-*co*-PEI_2_-*co*-PPyOx_13_) was further treated with acetic anhydride, and the mol % of PEI units as well as piperidine/pyrrolidine segments in the ionic polymer was reverse calculated from ^1^H NMR spectroscopy of the resultant copolymers after acetic anhydride treatment (Figures S8 and S10).

SEC traces of synthesized amine functional nonionic/ionic and copoly(2-oxazoline)s are unimodal and revealed relatively narrow molar mass distribution (*D̵* ≤ 1.20), as observed from SEC analysis (Figure 2B and Table 1). The apparently low molar mass (as observed from SEC) of copolymers compared to the theoretical value (Table 1) can be attributed to the decreased size of the polymer coil, further indicating enhanced polymer–polymer interactions in the DMA SEC eluent. It should be noted that we were unable to measure *M*_n_ and *D̵* of PEI_25_ and PEI_50_ from SEC because of their relatively low molar mass and insoluble nature in the DMA eluent.

The average molecular weight PEI_25_ and PEI_50_ could be determined from their respective ^1^H NMR spectrum (Figures S3 and S4) by calculating the integration ratio of the methylene protons (at δ 2.75 ppm) in the polymer backbone to the α-terminal methyl protons (at δ 2.41 ppm) and were found to be 1.2 (DP ∼ 27) and 2.28 (DP ∼ 27) kDa, respectively (Table 1). A similar molar mass/DP was also obtained by calculating the integration ratio of the methylene protons in the polymer backbone (at δ 2.75 ppm) to the ω-terminal methylene protons (δ 3.67 ppm) adjacent to the azide end group (Figure S3), further validating the DP calculation (Figure S3).

As can be seen from the turbidimetry plot in Figure 3, all newly synthesized copolymers were thermoresponsive and showed lower critical solution temperature-type (LCST-type) phase transition behavior in aqueous medium. LCST-type cloud points (*T*_CP_) were measured from the heating cycles and were found to be 28, 36, 50, 33, 61, 48, and 25 °C for PPiOx_27_-*co*-PPyOx_23_, PPiOx_13_-*co*-PPyOx_12_, PPiOx_10_-*co*-PEI_2_-*co*-PPyOx_13_, PPiOx_21_-*co*-PEI_4_, PPiOx_12_-*co*-PMoOx_13_, PPiOx_14_-*co*-PMoOx_11_, and PPiOx_20_-*co*-PMoOx_5_ copolymers, respectively. The thermal transition of all copolymers is relatively fast, and the copolymer exhibits very negligible hysteresis as the heating and, respectively, cooling turbidimetry curves are nearly identical (Figures S20–S26).

### KHI Performance Experiments

Some KHI high-pressure rocking cell tests were carried out with a 10 mL aqueous solution in each 40 mL cell rather than the 20 mL solution, which is usually used in our studies, including the initial previously reported study on poly(2-amino-2-oxazoline)s.^19^ The use of 10 mL of solution was due to limited amounts of some polymers being available. The volume of solution does slightly affect the *T*_o_ and *T*_a_ values, as has been reported previously.^24^ Thus, *T*_o_, and often also *T*_a_, values are lower with the increasing amount of aqueous KHI solution in the cell. For example, with 2500 ppm of a commercial VP/VCap copolymer, the average *T*_o_ value in the SCC tests at 76 bar SNG decreased from 8.2 °C with a 10 mL aqueous solution to 7.2 °C for the 20 mL solution. This is a difference on average (from 10 individual tests) of 1 °C, which was also statistically significant at the 95% confidence level from a statistical *t*-test (*p* < 0.095). The difference in *T*_o_ values at different aqueous volumes was suggested to be due to two factors, the water–gas interfacial area and the change in the thermodynamics of the system due to dissolved gases, including CO_2_.

In order to compare the new poly(2-amino-2-oxazoline)s (using 10 mL solution) to the polymers in the initial study (in which a 20 mL solution was used), some polymers were tested with both 10 and 20 mL of solution. Table 3 summarizes these results using SNG as the gas phase. The general conclusion agreed with earlier studies that decreasing the volume gave higher *T*_o_ and *T*_a_ values. As can be seen from the table, in this study, the average *T*_o_ value was about 0.5–1.0 °C lower for tests with 20 mL of KHI solution than for 10 mL of solution.

**Table 3:** Comparison of KHI Slow Constant Cooling Results in SNG for Varying Volume Aqueous Solutions^a^

	10 mL		20 mL	
Polymer	*T*_o_ (av.) (°C)	*T*_a_ (av.) (°C)	*T*_o_ (av.) (°C)	*T*_a_ (av.) (°C)
No additive	17.4	17.0	16.8	16.4
PVCap	10.3	9.8	9.7	9.3
PPiOx_27_-*co*-PPiOx_23_	10.1	9.8	9.4	8.9
			9.2	8.8
PPiOx_13_-*co*-PPyOx_12_	10.0	9.5	8.8	8.3
PPiOx_20_-*co*-PMoOx_5_	10.9	9.6	10.0	8.8

a *T*_o_ = macroscopic hydrate onset temperature, *T*_a_ = temperature at the start of the fastest gas hydrate growth. (Two sets of 5 tests were conducted for PPiOx_27_-*co*-PPiOx_23_.)

Table 4 summarizes the KHI test results using 10 mL of solution and 76 bar of SNG. As we have previously shown that the homopolymer PPyOx_25_ gave a worse KHI performance than the more hydrophobic PPiOx-*co*-PPyOx copolymer, we explored several new PPiOx–PPyOx copolymers. These were made in different ways, had variable molecular weights, and contained varying ratios of the 5-membered and 6-membered cyclic amine side chains. Some copolymers can give improved performance compared to homopolymers. For example, *N*-vinyl-*N*-methyl acetamide/*N*-vinyl caprolactam (VIMA/VCap) 1:1 copolymer and some 1-vinyl-3-alkylimidazolium bromide/VCap copolymers are more powerful KHIs than PVCap.^1,25,26^ This may be related to optimizing the surface-to weight ratio of the polymer to give maximum interaction with hydrate particle surfaces.^21^ Like VIMA/VCap, our copolymers are random (statistical) copolymers. There may be some benefit in making sequenced (e.g., ABABAB) copolymers as this would maximize the surface-to-weight ratio.^27,28^

**Table 4:** KHI Slow Constant Cooling Results with 10 mL of Solution in SNG^a^

Polymer	Polymer Concn (ppm)	Synergist Concn (ppm)	*T*_o_ (av.) (°C)	*T*_a_ (av.) (°C)
No additive			17.0	16.9
*i*BGE		5000	15.9	15.8
DPBGE		5000	15.4	15.3
PVCap	2500		10.3	9.8
PVCap + *i*BGE	2500	5000	6.8	4.1
PPiOx_14_-*co*-PPyOx_11_	2500		10.0	8.5
	5000		8.9	6.0
PPiOx_14_-*co*-PPyOx_11_ + *n*BGE	2500	5000	7.8	6.1
PPiOx_14_-*co*-PPyOx_11_ + *n*BGE	2500	10,000	7.7	6.0
PPiOx_14_-*co*-PPyOx_11_ + *i*BGE	2500	5000	7.3	5.0
PPiOx_14_-*co*-PPyOx_11_ + *i*BGE	2500	10,000	6.8	5.9
PPiOx_14_-*co*-PPyOx_11_ + DPBGE	2500	5000	7.5	6.1
PPiOx_27_-*co*-PPyOx_23_	10,000		8.1	5.9
	5000		7.8	7.1
	2500		10.1	9.8
	1000		12.2	12.0
PPiOx_27_-*co*-PPyOx_23_ + *i*BGE	2500	5000	9.0	8.8
PPiOx_13_-*co*-PPyOx_12_	10,000		8.5	6.2
	5000		8.6	7.5
	2500		10.0	9.5
	1000		12.4	12.1
PPiOx_10_-*co*-PEI_2_-*co*-PPyOx_13_	2500		10.9	10.5
PPiOx_21_-*co*-PEI_4_	2500		11.9	11.1
PPiOx_12_-*co*-PMoOx_13_	2500		11.1	10.3
	5000		9.9	9.0
PPiOx_12_-*co*-PMoOx_13_ + *i*BGE	5000	5000	9.4	8.5
PPiOx_14_-*co*-PMoOx_11_	2500		10.3	9.1
PPiOx_14_-*co*-PMoOx_11_ + *i*BGE	2500	5000	8.8	7.9
PPiOx_20_-*co*-PMoOx_5_	2500		10.9	9.6
	5000		8.3	6.7
PPiOx_20_-*co*-PMoOx_5_ + *i*BGE	5000	5000	7.0	4.8

a *T*_o_ = onset temperature, *T*_a_ = temperature at the start of the fastest hydrate growth.

At 2500 ppm, all of the PPiOx-*co*-PPyOx copolymers gave very similar KHI performance. The average *T*_o_ value was about 10.0–10.1 °C in all cases, even though the number of monomer units varied from 25 to 50. This is similar to the performance of commercial PVCap at 2500 ppm. The performance of KHI polymers depends on the molecular weight.^1,3^ Low molecular weight (1000–3000 g/mol or ca. 8–20 monomer units) is generally best for a monomodal weight distribution as it maximizes the surface-to-weight ratio at a given wt % concentration. The performance drops off gradually above this weight and drastically below this weight. Although the molecular weights of the polymers in our study are quite low, they are probably above the optimum weight for best performance, and the range of weight is also quite narrow. This may be the reason we do not observe clear differences in KHI performance due to the polymer molecular weight alone.

**Figure 6:**
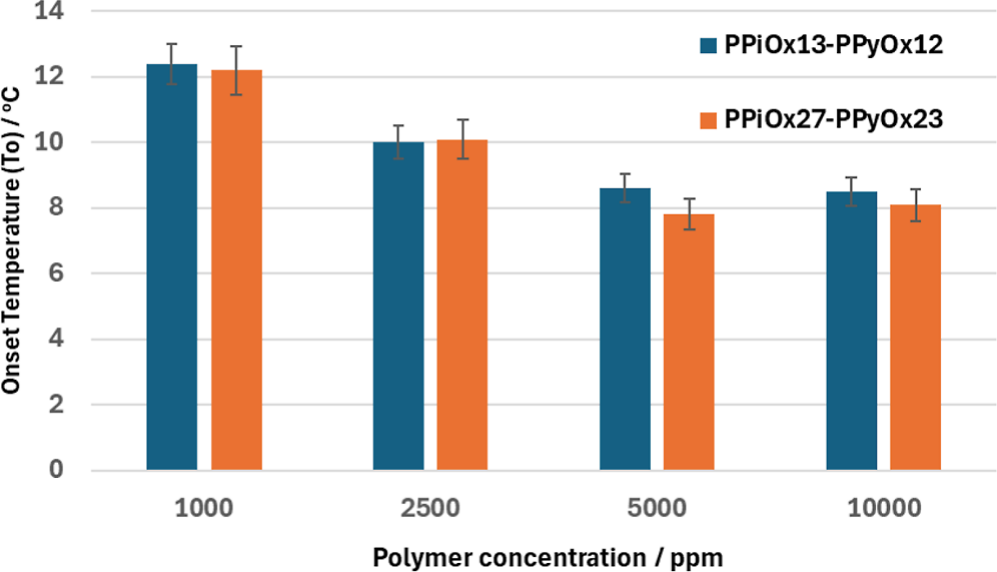
Effect of polymer concentration on average *T*_o_ values for two of the PPiOx–PPyOx copolymers using SNG.

The PPiOx-*co*-PPyOx copolymers were also tested at varying concentrations from 1000 to 10,000 ppm (Figure 6). In general, we see a good increase in performance, indicated by lowering of both *T*_o_ and *T*_a_ as the polymer concentration increased up to 5000 ppm but not beyond that at 10,000 ppm. This is a typical trend in SCC tests where the performance increase tends to tail off in the range 5000–10,000 ppm. For example, PPiOx_14_-*co*-PPyOx_11_ tested at 5000 ppm gave average *T*_o_/*T*_a_ values of 8.9/6.0 °C. This is a significant *T*_o_ value improvement compared to that at 2500 ppm of the same polymer, which had *T*_o_/*T*_a_ values of 10.0/8.5 °C. Furthermore, the average *T*_a_ value dropped even more than the *T*_o_ value, from 8.5 to 6.0 °C, indicating a strong ability of the polymer to slow down the gas hydrate growth rate.

The *T*_CP_ values of the PPiOx-*co*-PPiOx copolymers vary from 26 to 36 °C, which would limit their application in the field as explained in the Introduction section. Therefore, attempts were made to introduce more hydrophilic groups to raise the *T*_CP_, hopefully without losing KHI performance. This has been achieved previously for the VIMA/VCap 1:1 copolymer, which has a higher *T*_CP_ than PVCap as well as improved KHI performance.^25^ The more hydrophilic terpolymer PPiOx_10_-*co*-PEI_2_-*co*-PPyOx_13_ gave an average *T*_o_ of 10.9 °C at 2500 ppm, not much worse than those of the PPiOx-*co*-PPyOx copolymers. The advantage was that the copolymer had a *T*_CP_ of 50 °C. [PPiOx-*co*-PEI]_25_ with 10 mol % PEI, i.e., PPiOx_21_-*co*-PEI_4_, was also synthesized and tested. The *T*_CP_ was 32 °C and the average *T*_o_ was 11.9 °C, indicating no advantage over the PPiOx-*co*-PPyOx copolymers.

Given that PPiOx was a very active monomer in KHI polymers from the first study, we also investigated copolymers with the more hydrophilic PMoOx comonomer. Two of the three synthesized PPiOx-*co*-PMoOx copolymers also gave higher *T*_CP_ values than the PPiOx-*co*-PPyOx copolymers. PPiOx_12_-*co*-PMoOx_13_ and PPiOx_14_-*co*-PMoOx_11_ have cloud points of 61 and 48 °C, respectively. They both gave good KHI performance with average *T*_o_ values of 11.1 and 10.3 °C, respectively. The third copolymer, PPiOx_20_-*co*-PMoOx_5_, also gave a good KHI performance (*T*_o_ average = 10.9 °C), but the *T*_CP_ was only 24 °C. The performance of PPiOx_14_-*co*-PMoOx_11_ was not statistically significantly different from that of the best PPiOx–PPyOx copolymers, indicating that this copolymer is an improvement by giving a higher *T*_CP_ for a wider field application range. The performance is also similar to that of the commercial PVCap, which has a *T*_CP_ of 35 °C. We had enough of the PPiOx_20_-*co*-PMoOx_5_ copolymer to test at 5000 ppm. The performance improved significantly compared to that at 2500 ppm, with the average *T*_o_ value decreasing from 10.9 to 8.3 °C. The *T*_a_ value was also much lower than at 2500 ppm, giving 6.7 °C. In fact, none of the PPyOx copolymers gave immediate fast gas hydrate formation when *T*_o_ was reached, as judged by the *T*_o_–*T*_a_ values. This indicates that this class of KHI polymers have the ability to slow down the crystal growth. This is a useful property, giving the operator time to react when the first sign of hydrate formation is discovered.

Table 4 also gives results of solvent synergists that were tested with some of the poly(2-amino-2-oxazoline)s. We investigated the effect of the high flash point glycol ethers *n*-butyl glycol ether (*n*BGE), isobutyl glycol ether (*i*BGE), and dipropylenebutylglycol ether (DPBGE). *n*BGE is used in some commercial KHI formulations.^29−34^ These solvents are known to be better synergists at low concentrations (≤1 wt %) for PVCap and poly(*N*-isopropylmethacrylamide) than monoethylene glycol. This is due to the optimum size of the alkyl groups, which can give better interaction with hydrate surfaces than MEG or small alkyl groups.^21,29^ The PPiOx_14_-*co*-PPyOx_11_ copolymer was most comprehensively tested with the synergist solvents. Addition of 5000 ppm of any of the three solvents improved the KHI performance significantly. With added 5000 ppm of *n*BGE, the average *T*_o_ was lowered by 2.2 °C, from 10.0 °C down to 7.8 °C. There was no improvement when a further 5000 ppm (i.e., total 10,000 ppm of *n*BGE) was added. However, *i*BGE proved to give more synergy. With 5000 ppm of *i*BGE, the *T*_o_ value decreased from 10.0 to 7.3 °C and dropped further to 6.8 °C with 10,000 ppm of *i*BGE. Addition of 5000 ppm of DPBGE decreased the average *T*_o_ value to 7.5 °C. The *T*_a_ values also dropped considerably for all solvent tests compared to the test with the pure PPiOx_14_-*co*-PPyOx_11_ polymer. The glycol *i*BGE was investigated with some of the other polymers, as given in Table 4. In all cases, synergy was observed. For 2500 ppm of the larger copolymer PPiOx_27_-*co*-PPyOx_23_, the synergy was not as pronounced as 5000 ppm *i*BGE lowered *T*_o_ by only 1.1 °C. For 5000 ppm PPiOx_20_-*co*-PMoOx_5_, addition of 5000 ppm decreased the average *T*_o_ from 8.3 to 7.0 °C. In the first study, only one synergist solvent was investigated with one polymer, PPyOx_25_ homopolymer.^19^ 2500 ppm of this polymer gave a rather average *T*_o_ value of 11.1 °C. Addition of 5000 ppm *i*BGE decreased the *T*_o_to 9.2 °C. Note that this first study used tests with a 20 mL aqueous solution, and it must be remembered that these values would probably be 0.5–1.0 °C higher for tests with 10 mL polymer solution.

We also carried out KHI experiments in SNG with a 3 wt % NaCl solution with the polymers for which we had sufficient amount for several cell tests. 3 wt % NaCl lowers the hydrate equilibrium temperature by about 1.1 °C.^3,11,15^Table 5 summarizes the results. In general, we observed a small decrease in the average *T*_o_ values, which is assumed to be due to the thermodynamic effect of the added salt. Interestingly, the *T*_a_ values dropped more than the *T*_o_ values with the added salt, indicating that the salt improves the ability to arrest the hydrate crystal growth. This was also observed with PVCap.

**Table 5:** Comparison of KHI Polymer Tests Using SNG with and without 3 wt % Aqueous Sodium Chloride

Polymer	[Polymer] (ppm)	3% NaCl (ppm)	*T*_o_ (av.) (°C)	*T*_a_ (av.) (°C)
No additive			17.0	16.9
PVCap 10 mL	2500		10.3	9.8
		yes	8.9	7.5
PPiOx_13_-*co*-PPyOx_12_	2500		10.0	9.5
		yes	9.5	7.6
PPiOx_20_-*co*-PMoOx_5_	2500		10.9	9.6
		yes	8.2	7.1
PPiOx_10_-*co*-PEI_2_-*co*-PPyOx_13_	2500		10.9	10.5
		yes	10.6	8.4

Table 6 summarizes the KHI test results with 110 bar of methane as the gas phase. In general, the lowering of the *T*_o_ and *T*_a_ values compared to that with no additive was less than that for equivalent tests with SNG. It is well-known that the best KHIs do not perform well to the same level of subcooling with methane-rich gases as with the more common natural gas mixtures, i.e., so-called sI versus sII forming gases. Thus, compared to no additive, 2500 ppm of PVCap lowered the average *T*_o_ value by 4.3 °C for SNG (Table 6) and 6.7 °C for methane. Poly(2-amino-2-oxazoline)s also showed the same trend. Among the five polymers tested, PPiOx_13_-*co*-PPyOx_12_ gave the best results. The average *T*_o_ at 2500 ppm was 7.8 °C, which was not significantly different from the result for PVCap (8.0 °C). At 5000 ppm, PVCap showed slightly better performance than PPiOx_13_-*co*-PPyOx_12_, giving *T*_o_ values of 6.8 and 7.4 °C respectively. The higher molecular weight polymer PPiOx_27_–PPiOx_23_ did not perform as well as PPiOx_13_-*co*-PPyOx_12_, but we had enough polymer to carry out a test with added 5000 ppm *i*BGE. The glycol ether showed reasonable synergy, lowering the average *T*_o_ value from 9.9 to 8.5 °C. Interestingly, *i*BGE did not give as good synergy with either this polymer or PVCap as compared to tests with SNG (Table 4). PPiOx_27_–PPiOx_23_ also showed a typical trend with carrying concentration giving decreasing average *T*_o_ values when tested at 1000, 2500, and 5000 ppm.

**Table 6:** KHI Slow Constant Cooling Tests with 110 bar of Methane and 10 mL Solution^a^

Polymer	[Polymer] (ppm)	[Synergist] (ppm)	*T*_o_ (av.) (°C)	*T*_a_ (av.) (°C)
No additive			12.3	11.9
PVCap 10k	2500		8.0	7.4
	5000		6.8	5.9
PVCap 10k + *i*BGE	2500	5000	7.3	6.5
PPiOx_13_-*co*-PPyOx_12_	2500		7.8	7.3
	5000		7.4	6.3
PPiOx_13_-*co*-PPyOx_12_ + *i*BGE	2500	5000	7.2	6.2
PPiOx_27_-*co*-PPiOx_23_	1000		10.2	9.9
	2500		9.9	8.4
	5000		8.2	7.0
PPiOx_27_-*co*-PPiOx_23_ + *i*BGE	2500	5000	8.5	8.4
PPiOx_12_-*co*-PMoOx_13_	2500		8.6	8.0
PPiOx_14_-*co*-PMoOx_11_	2500		8.8	7.9
PPiOx_20_-*co*-PMoOx_5_	2500		8.6	7.6
	5000		6.7	5.6

a *T*_o_ = onset temperature, *T*_a_ = temperature at the start of the fastest hydrate growth.

The three PPiOx-*co*-PMoOx copolymers gave very similar results (average *T*_o_ = 8.6–8.7 °C), only slightly worse than that of PVCap at 2500 ppm. PPiOx_20_-*co*-PMoOx_5_ was also tested at 5000 ppm and gave the best performance of the five poly(2-amino-2-oxazoline)s tested at this concentration, equal to the performance of PVCap.

## Conclusions

A series of poly(2-amino-2-oxazoline) copolymers with structural variations were prepared and tested for their KHI performance in high-pressure rocking cells using the slow (1 °C/h) constant cooling method and both SNG and methane gas. Due to the amounts of polymers available, we used a 10 mL aqueous solution in the 40 mL steel cells rather than the 20 mL we commonly use and was also used in our previous study. In comparison tests, it was found that tests at 10 mL gave 0.5–1.0 °C higher *T*_o_ and *T*_a_ values than tests with 20 mL, in line with an earlier study. The PPiOx–PPiOx copolymers gave comparable performance to PVCap in both gas systems. Higher cloud point temperature (*T*_CP_ = 48–61 °C) polymers such as PPiOx_10_-*co*-PEI_2_-*co*-PPyOx_13_ and two PPiOx_20_-*co*-PMoOx copolymers also gave good performance but with marginally higher *T*_o_ values than the best PPiOx-*co*-PPiOx copolymers. Several butylated, high flash synergist solvents were identified that lowered the *T*_o_ and *T*_a_ values by about 2–3 °C relative to the polymer alone.

## Supporting Information Available

Additional experimental information including the materials and synthesis of all compounds, ^1^H NMR of all compounds (monomers and polymers), SEC traces of all polymers, and polymer turbidity measurements (PDF)

## References

[ref1] Kelland M. A. A review of kinetic hydrate inhibitors: Tailormade water-soluble polymers for oil and gas industry applications. In Advances in Materials Science Research; Wytherst M. C., Ed.; Nova Science Publishers, Inc: New York, 2011; Vol. 8.

[ref2] Perrin A.; Musa O. M.; Steed J. W. The chemistry of low dosage clathrate hydrate inhibitors. Chem. Soc. Rev. 2013, 42, 1996–2015. 10.1039/c2cs35340g. 23303391

[ref3] Kelland M. A. Production Chemicals for the Oil and Gas Industry, 2nd ed.; CRC Press: Boca Raton, FL, 2014.

[ref4] Zhukov A. Y.; Stolov M. A.; Varfolomeev M. A. Use of Kinetic Inhibitors of Gas Hydrate Formation in Oil and Gas Production Processes: Current State and Prospects of Development. Chem. Technol. Fuels Oils 2017, 53, 377–381. 10.1007/s10553-017-0814-6.

[ref5] Shahnazar S.; Bagheri S.; TermehYousefi A.; Mehrmashhadi J.; Abd Karim M. S.; Kadri N. A. Structure, mechanism, and performance evaluation of natural gas hydrate kinetic inhibitors. Rev. Inorg. Chem. 2018, 38, 1–19. 10.1515/revic-2017-0013.

[ref6] Kamal M. S.; Hussein I. A.; Sultan A. S.; von Solms N. Application of various water soluble polymers in gas hydrate inhibition. Energy Rev. 2016, 60, 206–225. 10.1016/j.rser.2016.01.092.

[ref7] Wang Y.; Fan S.; Lang X. Reviews of gas hydrate inhibitors in gas-dominant pipelines and application of kinetic hydrate inhibitors in China. Chin. J. Chem. Eng. 2019, 27, 2118–2132. 10.1016/j.cjche.2019.02.023.

[ref8] Chin Y. D.; Srivastava A. Advances in LDHIs and Applications, OTC-28905. In Offshore Technology Conference, Houston, Texas, USA, 30 April–3 May, 2018.

[ref9] Singh A.; Suri A. Review of Kinetic Hydrate Inhibitors Based on Cyclic Amides and Effect of Various Synergists. Energy Fuels 2021, 35 (19), 15301–15338. 10.1021/acs.energyfuels.1c02180.

[ref10] Singh A.; Suri A. A review on gas hydrates and kinetic hydrate inhibitors based on acrylamides. J. Nat. Gas Sci. Eng. 2020, 83, 103539 10.1016/j.jngse.2020.103539.

[ref11] Sloan E. D. Jr.; Koh C. A. Clathrate Hydrates of Natural Gases, 3rd ed.; CRC Press: Boca Raton, FL, 2008.

[ref12] Koh C. A.; Sum A. K.; Sloan E. D. Gas hydrates: Unlocking the energy from icy cages. J. Appl. Phys. 2009, 106, 061101 10.1063/1.3216463.

[ref13] Talley L. D.; Mitchell G. F. Application of Proprietary Kinetic Hydrate Inhibitors in Gas Flowlines. In Proceedings of the 1999 Offshore Technology Conference, Houston, TX, May 3–6, 1999; p OTC-11036-MS.

[ref14] Koh C. A. Towards a fundamental understanding of natural gas hydrates. Chem. Soc. Rev. 2002, 31 (3), 157–167. 10.1039/b008672j. 12122641

[ref15] Makogon T. Y. Handbook of Multiphase Flow Assurance; Elsevier Science & Technology, 2019.

[ref16] Kelland M. A. History of the development of low dosage hydrate inhibitors. Energy Fuels 2006, 20, 825–847. 10.1021/ef050427x.

[ref17] Aminnaji M.; Anderson R.; Hase A.; Tohidi B. Can kinetic hydrate inhibitors inhibit the growth of pre-formed gas hydrates?. Gas Sci. Eng. 2023, 109, 104831 10.1016/j.jngse.2022.104831.

[ref18] Luna-Ortiz E.; Healey M.; Anderson R.; Sørhaug E. Crystal Growth Inhibition Studies for the Qualification of a Kinetic Hydrate Inhibitor under Flowing and Shut-In Conditions. Energy Fuels 2014, 28 (5), 2902–2913. 10.1021/ef402493x.

[ref19] Kelland M. A.; Jana S.; Pomicpic J.; Sedlacek O.; Hoogenboom R. Kinetic Hydrate Inhibition from Thermoresponsive Poly(2-amino-2-oxazoline)s: Size and Shape of the Hydrophobic Groups Are Critical for Performance. Energy Fuels 2024, 38, 3784–3791. 10.1021/acs.energyfuels.3c05107.

[ref20] Sedlacek O.; Bera D.; Hoogenboom R. Poly(2-amino-2-oxazoline)s: a new class of thermoresponsive polymers. Polym. Chem. 2019, 10 (34), 4683–4689. 10.1039/C9PY00943D.

[ref21] Dirdal E. G.; Kelland M. A. Does the Cloud Point Temperature of a Polymer Correlate with Its Kinetic Hydrate Inhibitor Performance?. Energy Fuels 2019, 33, 7127–7137. 10.1021/acs.energyfuels.9b01185.

[ref22] Kelland M. A.; Dirdal E. G.; Pomicpic J.; Ajiro H.; Nag A. Kinetic Hydrate Inhibitors: The Effect of Pre- or Postpolymerization Solvent Addition on Performance and a Powerful New Glycol Ether Solvent Synergist. Energy Fuels 2023, 37, 11853–11863. 10.1021/acs.energyfuels.3c02054.

[ref23] Walpole R. E.; Myers R. H.; Myers S. L.; Walpole R. E.; Ye K. Probability and Statistics for Engineers and Scientists, 8th ed.; Pearson Education: Upper Saddle River, NJ, USA, 2007.

[ref24] Lone A.; Kelland M. A. Exploring Kinetic Hydrate Inhibitor Test Methods and Conditions Using a Multicell Steel Rocker Rig. Energy Fuels 2013, 27, 2536–2547. 10.1021/ef400321z.

[ref25] Colle K. S.; Oelfke R. H.; Kelland M. A. U.S. Patent 5,874,660 A, February 23, 1999.

[ref26] Rebolledo-Libreros M. E.; Reza J.; Trejo A.; Guzmán-Lucero D. J. Evaluation of copolymers from 1-vinyl-3-alkylimidazolium bromide and N-vinylcaprolactam as inhibitors of clathrate hydrate formation. J. Nat. Gas Sci. Eng. 2017, 40, 114–125. 10.1016/j.jngse.2017.02.008.

[ref27] Pomicpic J.; Kelland M. A.; Ghosh R.; Undheim A. Multi-functional Flow Assurance Chemicals: Corrosion and Kinetic Hydrate Inhibition from Maleic Anhydride:N-Vinyl Caprolactam Copolymers and Synergists. Energy Fuels 2023, 37, 8964–8975. 10.1021/acs.energyfuels.3c00991.

[ref28] Kelland M. A.; Koyama Y.; Pomicpic J.; Shinoda T. Kinetic Gas Hydrate Inhibition by Alternating Dipeptoids with Optimal Size and Shape N-Substituents. ACS Omega 2024, 9, 35475–35481. 10.1021/acsomega.4c02214. 39184499 PMC11340003

[ref29] Ree L. H. S.; Kelland M. A. Investigation of Solvent Synergists for Improved Kinetic Hydrate Inhibitor Performance of Poly(N-isopropyl methacrylamide). Energy Fuels 2019, 33 (9), 8231–8240. 10.1021/acs.energyfuels.9b01708.

[ref30] Mozaffar H.; Anderson R.; Tohidi B. Effect of alcohols and diols on PVCap-induced hydrate crystal growth patterns in methane systems. Fluid Phase Equilib. 2016, 425, 1–8. 10.1016/j.fluid.2016.05.005.

[ref31] Thieu V.; Bakeev K. N.; Shih J. S. Method for preventing or retarding the formation of gas hydrates. U.S. Patent 6,451,891 B1, 2002.

[ref32] Kelland M. A.; Dirdal E. G.; Ree L. H. S. Solvent Synergists for Improved Kinetic Hydrate Inhibitor Performance of Poly (N-vinyl caprolactam). Energy Fuels 2020, 34, 1653–1663. 10.1021/acs.energyfuels.9b03994.

[ref33] Cohen J. M.; Young W. D. Method for inhibiting the formation of gas hydrates. U.S. Patent 6,096,815 A, 2000.

[ref34] Dirdal E. G.; Kelland M. A. Further Investigation of Solvent Synergists for Improved Performance of Poly (N-vinylcaprolactam)-Based Kinetic Hydrate Inhibitors. Energy Fuels 2021, 35, 20103–20116. 10.1021/acs.energyfuels.1c03567.

[ref35] Mees M. A.; Hoogenboom R. Functional Poly(2-oxazoline)s by Direct Amidation of Methyl Ester Side Chains. Macromolecules 2015, 48 (11), 3531–3538. 10.1021/acs.macromol.5b00290.

[ref36] Sedlacek O.; Janouskova O.; Verbraeken B.; Hoogenboom R. Straightforward Route to Superhydrophilic Poly(2-oxazoline)s via Acylation of Well-Defined Polyethylenimine. Biomacromolecules 2019, 20 (1), 222–230. 10.1021/acs.biomac.8b01366. 30512933

[ref37] Van Guyse J. F. R.; Mees M. A.; Vergaelen M.; Baert M.; Verbraeken B.; Martens P. J.; Hoogenboom R. Amidation of methyl ester side chain bearing poly(2-oxazoline)s with tyramine: a quest for a selective and quantitative approach. Polym. Chem. 2019, 10 (8), 954–962. 10.1039/C9PY00014C.

[ref38] Van Guyse J. F. R.; Xu X.; Hoogenboom R. Acyl guanidine functional poly(2-oxazoline)s as reactive intermediates and stimuli-responsive materials. J. Polym. Sci., Part A: Polym. Chem. 2019, 57, 2616–2624. 10.1002/pola.29542.

