# Supplementary material for: Structural Design of Poly(2-amino-2-oxazoline)s for Kinetic Hydrate Inhibition of Natural Gas and Methane Hydrates
Source: ACS Omega. 2025 Apr 29;10(18):18690–9. doi: 10.1021/acsomega.5c00143 (PMC12079221; doi:10.1021/acsomega.5c00143)
Supplement: Supplementary file 1 — ao5c00143_si_001.pdf [file ao5c00143_si_001.pdf]

Structural design of poly(2-amino-2-oxazoline)s for kinetic hydrate inhibition of natural gas and methane hydrates

Malcolm A. Kelland,*[1] Somdeb Jana[2] , Julie Kiær,[1] Ajla Salihovic[1] Janronel Pomicpic,[1] and Richard Hoogenboom[2]*

[1] Department of Chemistry, Bioscience and Environmental Engineering, Faculty of Science and Technology, University of Stavanger, N-4036 Stavanger, Norway

[2] Supramolecular Chemistry Group, Krijgslaan 281 - Building S4, B-9000, Ghent, Belgium

Supporting Information

Experimental section

Materials

2-Ethyl-2-oxazoline (EtOx, kindly donated by Polymer Chemistry Innovations (PCI)), methyl *p*-toluenesulfonate (MeOTs, 98%, Merck) and acetonitrile (ACN, Merck) were purified by distillation over anhydrous barium oxide (BaO) and stored under inert atmosphere in a glove box. Extra dry *N,N*-dimethyl acetamide and *N,N*-dimethyl formamide (Acroseal, stored over molecular sieves, 99.85%) were purchased from Fisher scientific and used as received. Triethylamine (Et_3N, 98%, Merck) was purified over aluminum oxide using a solvent purification system from J.C. Meyer. All other chemicals were used as received including: 1-pyrrolidinecarbonyl chloride (Merck, 97%), 1-piperidinecarbonyl chloride (Merck, 97%), 4-morpholinecarbonyl chloride (Merck, 98 %), hydrogen chloride (HCl, 37wt%, Fisher scientific), anhydrous barium oxide (BaO, 99.99% trace metal basis, Merck), ultra-dry sodium azide (NaN_3, >99%, Merck), acetic anhydride (99.5%, Merck).

Synthesis of poly(2-ethyl-2-oxazoline) (PEtOx) homopolymer:

PEtOx with two different DP's (25 and 50) were synthesized by microwave-assisted CROP of EtOx at 140 °C in acetonitrile using MeOTs as an initiator and terminated with sodium azide according to a literature procedure.[1] Typically, in the glovebox, the monomer EtOx (6 mL, 59.43 mmol) and the MeOTs initiator (0.357 mL, 2.37 mmol, [EtOx]:[MeOTs] = 25:1) were dissolved in 9 mL dry ACN in a 10-20 mL microwave vial. The vial was then heated at 80 °C in microwave reactor for a predetermined time and cooled down to room temperature, followed by termination with solid NaN_3 (1.54 gm, 23.7 mmol) and stirring overnight. The GC analysis revealed > 90% conversion of the monomer. The reaction mixture was then filtered and precipitated in cold (-30 °C) diethyl ether and dried under reduced pressure at 40 °C. The crude polymer was dissolved in deionized water and purified by dialysis (regenerated cellulose membrane with a molecular weight cut-off of 100-500 Da) against deionized water followed by lyophilization to obtain PEtOx (Yield: 80%) as a white hygroscopic powder. Another PEtOx homopolymer with DP 50 was also prepared by varying the [EtOx] and [MeOTs] ratio to 50:1. ^{1}H NMR (300 MHz; CD_3OD) δ (ppm): 3.30-3.59 (4H, *br*, $NC\underline{H}_2C\underline{H}_2$), 2.18-2.46 (2H, *br*, $COC\underline{H}_2$) and 0.90-1.10 (3H, *br*, $COCH_2C\underline{H}_3$).

Synthesis of poly(ethylene imine) (PEI) homopolymer:

Linear polyethyleneimine (PEI) was synthesized according to the previously reported procedure.[2] PEtOx homopolymer (2.0 gm) was dissolved in aqueous hydrochloric acid (~18 wt %, 17 mL) and heated for 120 minutes at 120 °C in a microwave reactor. The solvents were then evaporated under reduced pressure at high temperature. The crude product was suspended in ice-cold water (100 mL) and ice-cold NaOH (2 M) was added dropwise into the suspension until it dissolved. Upon further addition of NaOH, the free base of PEI precipitated at pH 10-11. The precipitate was filtered, washed with ice-cold distilled water, recrystallized, and dried under high vacuum at room temperature to obtain PEI as a white powder (Yield: 65 %).

^{1}H NMR (300 MHz; CD_3OD) δ (ppm): 2.71 (4H, *br*, NHC$\underline{H}_2$C$\underline{H}_2$).

Synthesis of poly(2-piperidinyl-2-oxazoline)$_{27}$-*co*-poly(2-pyrrolidinyl-2-oxazoline)$_{23}$ (PPiOx$_{27}$-*co*-PPyOx$_{23}$):

Neutralized linear polyethyleneimine (PEI$_{50}$, 642 mg, 0.292 mmol, 1 equiv.) was suspended in dry 100 mL of dry, degassed *N,N*-dimethylacetamide (DMA) under an argon atmosphere. The reaction mixture was then cooled to ~ 0 °C using ice/water bath and triethylamine (6.5 mL, 44 mmol, 3 equiv. with respect to amine groups) was added slowly. Afterwards, 1-pyrrolidinecarbonyl chloride (0.970 mL, 8.76 mmol, 0.6 equiv. with respect to amine groups) and 1-piperidinecarbonyl chloride (0.900 mL, 7.3 mmol, 0.5 equiv. with respect to amine groups) were added dropwise and the reaction mixture was stirred at room temperature for 12 h. The solvent was removed under reduced pressure and the residue was dissolved in 40 mL of methanol/water mixture (3:1; v/v). The solution was transferred into dialysis tubes (with molecular weight cut-off of 0.1-0.5 kDa) and dialyzed against methanol/water mixture (2.5:1; v/v) for two days and finally with distilled water for another two days. Afterwards, the pure polymer was recovered by freeze-drying as colorless powdery solid. The full conversion of the amines and compositions of pyrrolidine and piperidine segments were confirmed by ^{1}H NMR spectroscopy.

^{1}H NMR (300 MHz; CD_3OD) δ (ppm): 1.73 (4H, *br*, -CH$_2$C$\underline{H}_2$C$\underline{H}_2$CH$_2$-; from pyrrolidine moiety), 1.48 (6H, *br*, -CH$_2$C$\underline{H}_2$C$\underline{H}_2$C$\underline{H}_2$CH$_2$-; from piperidine moiety), 3.02 (4H, *br*, -C$\underline{H}_2$CH$_2$CH$_2$C$\underline{H}_2$- from pyrrolidine as well as 4H, *br*, -C$\underline{H}_2$CH$_2$CH$_2$CH$_2$C$\underline{H}_2$- from piperidine), 3.26 (4H, *br*, -NC$\underline{H}_2$C$\underline{H}_2$N-).

Synthesis of poly(2-piperidinyl-2-oxazoline)$_{13}$-*co*-poly(2-pyrrolidinyl-2-oxazoline)$_{12}$ (PPiOx$_{13}$-*co*-PPyOx$_{12}$):

Linear polyethyleneimine (PEI_{25}, 620 mg, 0.5 mmol, 1 equiv.) was suspended in dry 100 mL of dry, degassed *N,N*-dimethylacetamide (DMA) under an argon atmosphere. The reaction mixture was then cooled to ~ 0 °C using ice/water bath and triethylamine (6 mL, 40 mmol, 3 equiv. with respect to amine groups) was added slowly. Afterwards, 1-pyrrolidinecarbonyl chloride (0.950 mL, 8.3 mmol, 0.6 equiv. with respect to amine groups) and 1-piperidinecarbonyl chloride (0.820 mL, 7.0 mmol, 0.5 equiv. with respect to amine groups) were added dropwise and the reaction mixture was stirred at room temperature for 12 h. The solvent was removed under reduced pressure and the residue was dissolved in 40 mL of methanol/water mixture (3:1; v/v). The solution was transferred into dialysis tubes (with molecular weight cut-off of 0.1-0.5 kDa) and dialyzed against methanol/water mixture (2.5:1; v/v) for two days and finally with distilled water for another two days. Afterwards, the pure polymer was recovered by freeze-drying as colorless powdery solid. The full conversion of the amines and compositions of pyrrolidine and piperidine segments were confirmed by ^1H NMR spectroscopy.

^1H NMR (300 MHz; CD_3OD) δ (ppm): 1.85 (4H, *br*, $-CH_2C\underline{H}_2C\underline{H}_2CH_2-$; from pyrrolidine moiety), 1.65 (6H, *br*, $-CH_2C\underline{H}_2C\underline{H}_2C\underline{H}_2CH_2-$; from piperidine moiety), 3.15 (4H, *br*, $-C\underline{H}_2CH_2CH_2C\underline{H}_2-$ from pyrrolidine as well as 4H, *br*, $-C\underline{H}_2CH_2CH_2CH_2C\underline{H}_2-$ from piperidine), 3.31 (4H, *br*, $-NC\underline{H}_2C\underline{H}_2N-$).

Synthesis of poly(2-piperidinyl-2-oxazoline)$_{10}$-*co*-poly(ethyleneimine)$_2$-*co*-poly(2-pyrrolidinyl-2-oxazoline)$_{13}$ (PPiOx$_{10}$-*co*-PEI$_2$-*co*-PPyOx$_{13}$):

Linear polyethyleneimine (PEI_{25}, 700 mg, 0.580 mmol, 1 equiv.) was suspended in dry 100 mL of dry, degassed *N,N*-dimethylacetamide (DMA) under an argon atmosphere. The reaction mixture was then cooled to ~ 0 °C using ice/water bath and triethylamine (7.5 mL, 53 mmol, 3 equiv. with respect to amine groups) was added slowly. Afterwards, 1-pyrrolidinecarbonyl

chloride (0.860 mL, 7.87 mmol, 0.5 equiv. with respect to amine groups) and 1-piperidinecarbonyl chloride (0.700 mL, 6.25 mmol, 0.38 equiv. with respect to amine groups) were added dropwise and the reaction mixture was stirred at room temperature for 12 h. The solvent was removed under reduced pressure at ambient temperature and the residue was dissolved in 40 mL of methanol/water mixture (3:1; v/v). The solution was transferred into dialysis tubes (with molecular weight cut-off of 0.1-0.5 kDa) and dialyzed against methanol/water mixture (2.5:1; v/v) for two days and finally with distilled water for another two days. Afterwards, the pure polymer was recovered by freeze-drying as colorless powdery solid. The compositions of pyrrolidine and piperidine segments were confirmed by ^{1}H NMR spectroscopy. A few mg of isolated polymer was further treated with excess of acetic anhydride and the mol% of free PEI units in the precursor polymer was estimated from ^{1}H NMR spectroscopy of the subsequent isolated polymer.

^{1}H NMR (300 MHz; CD_3OD) δ (ppm): 1.85 (4H, *br*, -$CH_2C\underline{H}_2C\underline{H}_2CH_2$-; from pyrrolidine moiety), 1.65 (6H, *br*, -$CH_2C\underline{H}_2C\underline{H}_2C\underline{H}_2CH_2$-; from piperidine moiety), 3.16 (4H, *br*, -$C\underline{H}_2CH_2CH_2C\underline{H}_2$- from pyrrolidine as well as 4H, *br*, -$C\underline{H}_2CH_2CH_2CH_2C\underline{H}_2$- from piperidine), 3.3 (4H, *br*, -$NC\underline{H}_2C\underline{H}_2N$-).

Synthesis of poly(2-piperidinyl-2-oxazoline)$_{21}$-*co*-poly(ethyleneimine)$_4$ (PPiOx$_{21}$-*co*-PEI$_4$):

Linear polyethyleneimine (PEI$_{25}$, 580 mg, 0.48 mmol, 1 equiv.) was suspended in dry 100 mL of dry, degassed *N,N*-dimethylacetamide (DMA) under an argon atmosphere. The reaction mixture was then cooled to ~ 0 °C using ice/water bath and triethylamine (6.0 mL, 40 mmol, 3 equiv. with respect to amine groups) was added slowly. Afterwards, 1-piperidinecarbonyl chloride (1.30 mL, 10.4 mmol, 0.8 equiv. with respect to amine groups) were added dropwise and the reaction mixture was stirred at room temperature for 12 h. The solvent was removed under reduced pressure at ambient temperature and the residue was dissolved in 40 mL of

methanol/water mixture (3:1; v/v). The solution was transferred into dialysis tubes (with molecular weight cut-off of 0.1-0.5 kDa) and dialyzed against methanol/water mixture (2.5:1; v/v) for two days and finally with distilled water for another two days. Afterwards, the pure polymer was recovered by freeze-drying as colorless powdery solid. The compositions of pyrrolidine and piperidine segments were confirmed by ^{1}H NMR spectroscopy. A few mg of isolated polymer was further treated with excess of acetic anhydride and the mol% of free PEI units as well as piperidine segments in the precursor polymer were estimated from ^{1}H NMR spectroscopy of the subsequent isolated polymer.

^{1}H NMR (300 MHz; CD_3OD) δ (ppm): 1.48 (6H, *br*, $-CH_2C\underline{H}_2C\underline{H}_2C\underline{H}_2CH_2-$; from piperidine moiety), 3.02 (4H, *br*, $-C\underline{H}_2CH_2CH_2CH_2C\underline{H}_2-$ from piperidine), 3.26 (4H, *br*, $-NC\underline{H}_2C\underline{H}_2N-$).

Synthesis of poly(2-piperidinyl-2-oxazoline)$_{12}$-*co*-poly(2-morpholinyl-2-oxazoline)$_{13}$ (PPiOx$_{12}$-*co*-PMoOx$_{13}$):

Linear polyethyleneimine (PEI$_{25}$, 550 mg, 0.450 mmol, 1 equiv.) was suspended in dry 100 mL of dry, degassed *N,N*-dimethylacetamide (DMA) under an argon atmosphere. The reaction mixture was then cooled to ~ 0 °C using ice/water bath and triethylamine (6.0 mL, 40 mmol, 3 equiv. with respect to amine groups) was added slowly. Afterwards, 4-morpholinecarbonyl chloride (0.71 mL, 6.1 mmol, 0.50 equiv. with respect to amine groups) and 1-piperidinecarbonyl chloride (1.0 mL, 8.0 mmol, 0.65 equiv. with respect to amine groups) were added dropwise and the reaction mixture was stirred at room temperature for 12 h. The solvent was removed under reduced pressure and the residue was dissolved in 40 mL of methanol/water mixture (3:1; v/v). The solution was transferred into dialysis tubes (with molecular weight cut-off of 0.1-0.5 kDa) and dialyzed against methanol/water mixture (2.5:1; v/v) for two days and finally with distilled water for another two days. Afterwards, the pure polymer was recovered by freeze-drying as colorless powdery solid. The full conversion of the amines and

compositions of morpholine and piperidine segments were confirmed by ^{1}H NMR spectroscopy.

^{1}H NMR (300 MHz; CD_3OD) δ (ppm): 3.61 (4H, *br*, $-C\underline{H}_2OC\underline{H}_2-$; from morpholine moiety), 1.50 (6H, *br*, $-CH_2C\underline{H}_2C\underline{H}_2C\underline{H}_2CH_2-$; from piperidine moiety), 3.06 (4H, *br*, $-C\underline{H}_2CH_2CH_2CH_2C\underline{H}_2-$ from piperidine as well as 4H, *br*, $-C\underline{H}_2NC\underline{H}_2-$ from morpholine), 3.25 (4H, *br*, $-NC\underline{H}_2C\underline{H}_2N-$).

Synthesis of poly(2-piperidinyl-2-oxazoline)$_{14}$-*co*-poly(2-morpholinyl-2-oxazoline)$_{11}$ (PPiOx$_{14}$-*co*-PMoOx$_{11}$):

Linear polyethyleneimine (PEI_{25}, 500 mg, 0.417 mmol, 1 equiv.) was suspended in dry 100 mL of dry, degassed *N,N*-dimethylacetamide (DMA) under an argon atmosphere. The reaction mixture was then cooled to ~ 0 °C using ice/water bath and triethylamine (6.0 mL, 40 mmol, 3 equiv. with respect to amine groups) was added slowly. Afterwards, 4-morpholinecarbonyl chloride (0.450 mL, 3.8 mmol, 0.35 equiv. with respect to amine groups) and 1-piperidinecarbonyl chloride (1.10 mL, 9.0 mmol, 0.8 equiv. with respect to amine groups) were added dropwise and the reaction mixture was stirred at room temperature for 12 h. The solvent was removed under reduced pressure and the residue was dissolved in 40 mL of methanol/water mixture (3:1; v/v). The solution was transferred into dialysis tubes (with molecular weight cut-off of 0.1-0.5 kDa) and dialyzed against methanol/water mixture (2.5:1; v/v) for two days and finally with distilled water for another two days. Afterwards, the pure polymer was recovered by freeze-drying as colorless powdery solid. The full conversion of the amines and compositions of morpholine and piperidine segments were confirmed by ^{1}H NMR spectroscopy.

^{1}H NMR (300 MHz; CD_3OD) δ (ppm): 3.71 (4H, *br*, $-C\underline{H}_2OC\underline{H}_2-$; from morpholine moiety), 1.65 (6H, *br*, $-CH_2C\underline{H}_2C\underline{H}_2C\underline{H}_2CH_2-$; from piperidine moiety), 3.10 (4H, *br*, -

$C\underline{H}_2CH_2CH_2CH_2C\underline{H}_2$- from piperidine as well as 4H, *br*, -$C\underline{H}_2NC\underline{H}_2$- from morpholine), 3.26 (4H, *br*, -$NC\underline{H}_2C\underline{H}_2N$-).

Synthesis of poly(2-piperidinyl-2-oxazoline)$_{20}$-*co*-poly(2-morpholinyl-2-oxazoline)$_5$ (PPiOx$_{20}$-*co*-PMoOx$_5$):

Linear polyethyleneimine (PEI$_{25}$, 410 mg, 0.341 mmol, 1 equiv.) was suspended in dry 100 mL of dry, degassed *N,N*-dimethylacetamide (DMA) under an argon atmosphere. The reaction mixture was then cooled to ~ 0 °C using ice/water bath and triethylamine (4.0 mL, 28 mmol, 3 equiv. with respect to amine groups) was added slowly. Afterwards, 4-morpholinecarbonyl chloride (0.11 mL, 1.01 mmol, 0.12 equiv. with respect to amine groups) and 1-piperidinecarbonyl chloride (1.3 mL, 10.36 mmol, 1.14 equiv. with respect to amine groups) were added dropwise and the reaction mixture was stirred at room temperature for 12 h. The solvent was removed under reduced pressure and the residue was dissolved in 40 mL of methanol/water mixture (3:1; v/v). The solution was transferred into dialysis tubes (with molecular weight cut-off of 0.1-0.5 kDa) and dialyzed against methanol/water mixture (2.5:1; v/v) for two days and finally with distilled water for another two days. Afterwards, the pure polymer was recovered by freeze-drying as colorless powdery solid. The full conversion of the amines and compositions of morpholine and piperidine segments were confirmed by ^{1}H NMR spectroscopy.

^{1}H NMR (300 MHz; CD_3OD) δ (ppm): 3.68 (4H, *br*, -$C\underline{H}_2OC\underline{H}_2$-; from morpholine moiety), 1.56 (6H, *br*, -$CH_2C\underline{H}_2C\underline{H}_2C\underline{H}_2CH_2$-; from piperidine moiety), 3.15 (4H, *br*, -$C\underline{H}_2CH_2CH_2CH_2C\underline{H}_2$- from piperidine as well as 4H, *br*, -$C\underline{H}_2NC\underline{H}_2$- from morpholine), 3.30 (4H, *br*, -$NC\underline{H}_2C\underline{H}_2N$-).

Synthesis of poly(2-pyrrolidinyl-2-oxazoline) ($PPyOx_{25}$):

The polymer was synthesized according to our previously reported protocol.[3] Typically, linear polyethyleneimine (PEI_{25}, 600 mg, 0.5581 mmol, 1 equiv.) was suspended in 100 mL of dry, degassed *N,N*-dimethylacetamide (DMA) under an argon atmosphere and cooled with an ice-water bath. Triethylamine (6 mL, 42 mmol, 3 equiv. with respect to amine groups) and 1-pyrrolidinecarbonyl chloride (5.0 mL, 42 mmol, 3 equiv. with respect to amine groups) were added dropwise and the reaction mixture was stirred at room temperature for 12 h. The solvent was removed under reduced pressure and the residue was dissolved in 40 mL of methanol/water mixture (3:1; v/v). The solution was transferred into a dialysis tube (with molecular weight cut-off of 0.1-0.5 kDa) and dialyzed against methanol/water mixture (2.5:1; v/v) for two days and finally with distilled water for another two days. Afterwards, the pure polymer was recovered by freeze-drying as colorless powdery solid. The full conversion of the amines was confirmed by 1H NMR spectroscopy.

^1H NMR (300 MHz; D_2O) δ (ppm): 1.65 (4H, *br*, $-CH_2C\underline{H}_2C\underline{H}_2CH_2-$), 3.12 (4H, *br*, $-C\underline{H}_2CH_2CH_2C\underline{H}_2-$), 3.21 (4H, *br*, $-NC\underline{H}_2C\underline{H}_2N-$).

Synthesis of poly(2-piperidinyl-2-oxazoline) ($PPiOx_{25}$):

The polymer was synthesized according to our previously reported protocol.[3] Typically, linear polyethyleneimine (PEI_{25}, 600 mg, 0.5581 mmol, 1 equiv.) was suspended in 100 mL of dry, degassed *N,N*-dimethylacetamide (DMA) under an argon atmosphere and cooled with an ice-water bath. Triethylamine (6 mL, 42 mmol, 3 equiv. with respect to amine groups) and 1-piperidinecarbonyl chloride (5.3 mL, 42 mmol, 3 equiv. with respect to amine groups) were added dropwise and the reaction mixture was stirred at room temperature for 12 h. The solvent was removed under reduced pressure and the residue was dissolved in 40 mL of methanol/water

mixture (3:1; v/v). The solution was transferred into a dialysis tube (with molecular weight cut-off of 0.1-0.5 kDa) and dialyzed against methanol/water mixture (2.5:1; v/v) for two days and finally with distilled water for another two days. Afterwards, the pure polymer was recovered by freeze-drying as colorless powdery solid. The full conversion of the amines was confirmed by 1H NMR spectroscopy.

^{1}H NMR (300 MHz; CD_3OD) δ (ppm): 1.49 (6H, *br*, $-CH_2C\underline{H_2}C\underline{H_2}C\underline{H_2}CH_2-$), 3.02 (4H, *br*, $-C\underline{H_2}CH_2CH_2CH_2C\underline{H_2}-$), 3.20 (4H, *br*, $-NC\underline{H_2}C\underline{H_2}N-$).

Synthesis of poly(2-piperidinyl-2-oxazoline)-*co*-poly(2-pyrrolidinyl-2-oxazoline) ($PPiOx_{14}$-*co*-$PPyOx_{11}$):

The polymer was synthesized according to our previously reported protocol.[3] Typically, linear polyethyleneimine (PEI_{25}, 550 mg, 0.423 mmol, 1 equiv.) was suspended in dry 100 mL of dry, degassed *N,N*-dimethylacetamide (DMA) under an argon atmosphere. The reaction mixture was then cooled to ~ 0 °C using ice/water bath and triethylamine (6 mL, 40 mmol, 3 equiv. with respect to amine groups) was added slowly. Afterwards, 1-pyrrolidinecarbonyl chloride (0.850 mL, 7.614 mmol, 0.6 equiv. with respect to amine groups) and 1-piperidinecarbonyl chloride (0.750 mL, 6.345 mmol, 0.5 equiv. with respect to amine groups) were added dropwise and the reaction mixture was stirred at room temperature for 12 h. The solvent was removed under reduced pressure and the residue was dissolved in 40 mL of methanol/water mixture (3:1; v/v). The solution was transferred into a dialysis tube (with molecular weight cut-off of 0.1-0.5 kDa) and dialyzed against methanol/water mixture (2.5:1; v/v) for two days and finally with distilled water for another two days. Afterwards, the pure polymer was recovered by freeze-drying as colorless powdery solid. The full conversion of the amines and compositions of pyrrolidine and piperidine segments were confirmed by ^{1}H NMR spectroscopy.

^{1}H NMR (300 MHz; CD_3OD) δ (ppm): 1.85 (4H, *br*, $-CH_2C\underline{H}_2C\underline{H}_2CH_2-$; from pyrrolidine moiety), 1.61 (6H, *br*, $-CH_2C\underline{H}_2C\underline{H}_2C\underline{H}_2CH_2-$; from piperidine moiety), 3.12 (4H, *br*, $-C\underline{H}_2CH_2CH_2C\underline{H}_2-$ from pyrrolidine as well as 4H, *br*, $-C\underline{H}_2CH_2CH_2CH_2C\underline{H}_2-$ from piperidine), 3.3 (4H, *br*, $-NC\underline{H}_2C\underline{H}_2N-$).

Methods:

Microwave polymerization: Cationic ring-opening polymerizations (CROP) were conducted in a single-mode microwave Biotage Initiator Sixty reactor (Biotage, Uppsala, Sweden) utilizing capped reaction vials. The reaction vials were heated to 200°C overnight, allowed to cool to room temperature under vacuum and filled with N_2 prior to use. All microwave polymerizations were performed with temperature control (IR sensor). Reaction mixtures for CROP were prepared in a VIGOR Sci-Lab SG 1200/750 Glovebox system, with 0 and <1 ppm of O_2 and H_2O, respectively.

Nuclear magnetic resonance (NMR). Bruker Avance 300 MHz Ultrashield or Bruker Avance II 400 MHz were used to measure ^{1}H-NMR spectra at room temperature. The chemical shifts (δ) are given in parts per million (ppm) relative to the solvent peak of D_2O (4.79 ppm) or CD_3OD (4.87; 3.31 ppm) as marked at each spectrum.

Size-exclusion chromatography (SEC): SEC was used to determine the molecular weights (M_w: weight-averaged molar mass; M_n: number-averaged molar mass) and dispersity ($Đ = M_w/M_n$) of the prepared polymers. The molar mass of $PEtOx_{25}$, $PEtOx_{50}$, $PPiOx_{25}$ homopolymers and $PPiOx_{27}$-*co*-$PPyOx_{23}$, $PPiOx_{13}$-*co*-$PPyOx_{12}$, $PPiOx_{10}$-*co*-PEI_2-*co*-$PPyOx_{13}$, $PPiOx_{21}$-*co*-PEI_4, $PPiOx_{12}$-co-$PMoOx_{13}$, $PPiOx_{14}$-co-$PMoOx_{11}$, $PPiOx_{20}$-co-$PMoOx_5$ copolymers were determined by SEC using an Agilent 1260-series HPLC system equipped with a 1260 online degasser, a 1260 ISO-pump, a 1260 automatic liquid sampler (ALS), a thermostatted column compartment (TCC) set at 50°C equipped with two PL gel 5 µm mixed-D columns (7.5 mm × 300 mm) and a precolumn in series, a 1260 diode array detector (DAD) and a 1260 refractive

index detector (RID). The used eluent was *N*,*N*-dimethyl acetamide (DMA) containing 50 mM of LiCl at a flow rate of 0.500 mL/min. The spectra were analyzed using the Agilent Chemstation software with the GPC add on. Molar mass values and molar mass distribution, i.e. dispersity (*Đ*) values were calculated against narrow disperse poly(methyl methacrylate) (PMMA) standards from PSS.

Another SEC system with aqueous medium as eluent was also employed to measure the molar mass distributions of $PPyOx_{25}$ homopolymer as the polymers are insoluble in DMA eluent. This was performed on an Agilent 1260-series HPLC system equipped with an online PSS degasser, a 1260 ISO-pump, a 1260 automatic liquid sampler (ALS), a 1261 thermostatic column compartment (TCC) at 35 °C equipped with two PSS Novema Max 5 µm columns and a precolumn in series, a 1262 diode array detector (DAD) and a 1290 refractive index detector (RID). The used eluent was methanol-sodium acetate buffer containing 0.1M $NaNO_3$ at a flow rate of 0.500 mL min^{-1}. The spectra were analyzed using the Agilent Chemstation software with the GPC add on. Molar mass and *Đ* values were calculated against PEG standards from PSS.

Cloud point (T_{cp}) measurements: The LCST-type cloud point temperatures (T_{cp}) of the amine functional (co)poly(2-oxazoline)s in distilled water were determined using Crystal 16™ parallel crystallizer turbidimeter (Avantium Technologies) connected to a recirculation chiller at a polymer concentration of 2 mg/mL and heating/cooling rate of 1.0 °C min^{-1}. The T_{cp} was reported as the temperature with 50% transmittance in the second heating run.

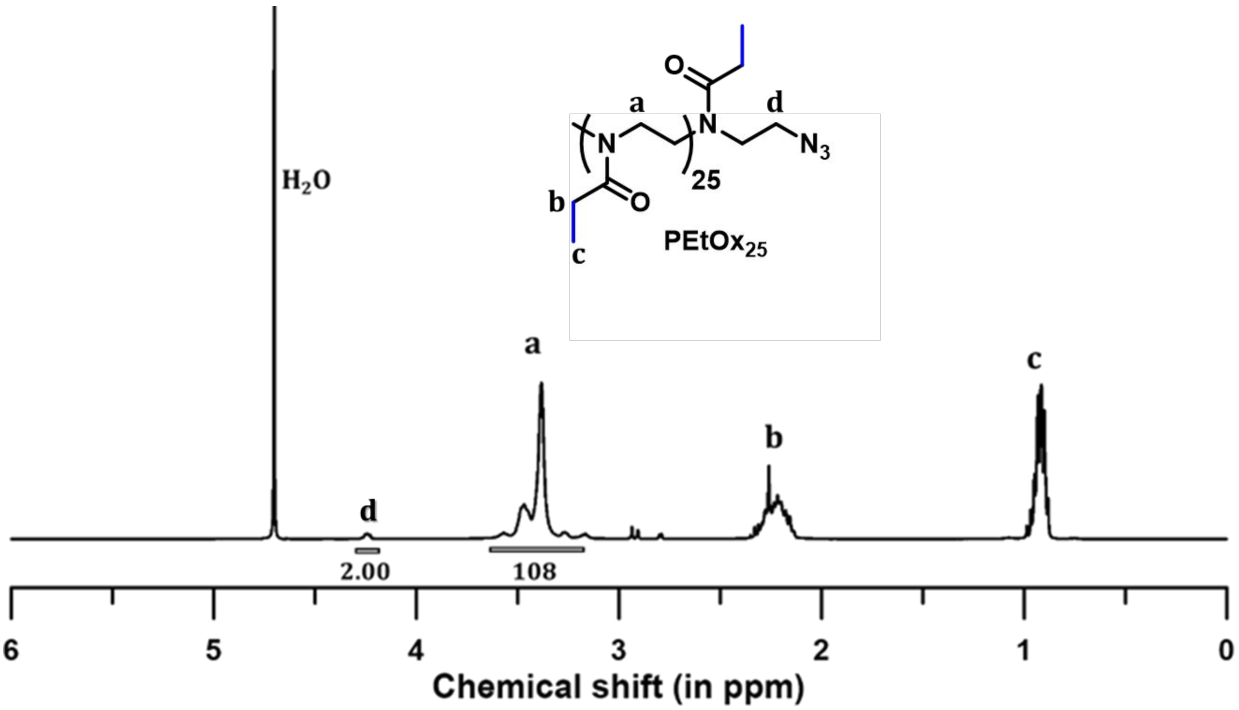

Figure S1. ^{1}H NMR spectrum of $PEtOx_{25}$ in D_2O.

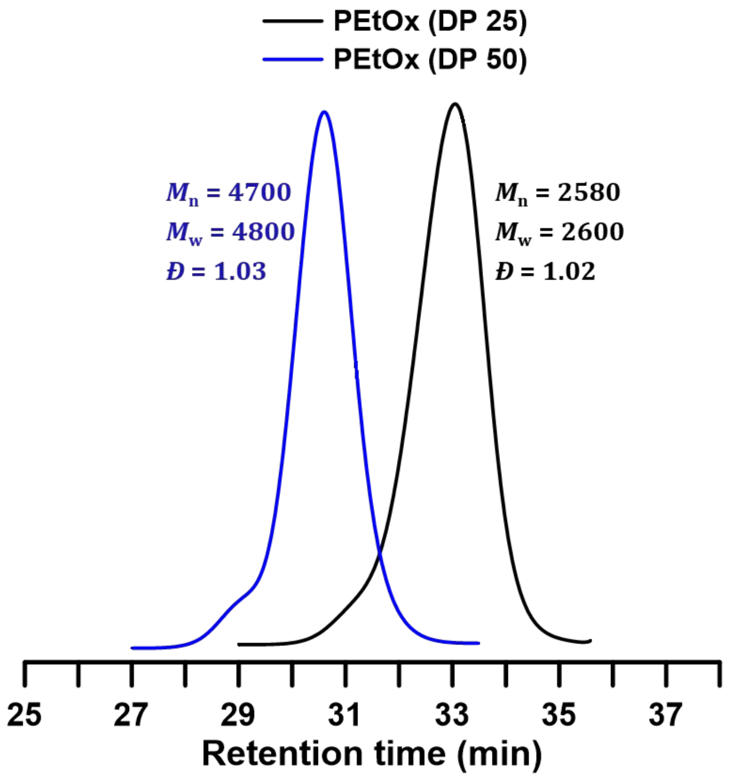

Figure S2. SEC traces of prepared $PEtOx_{25}$ and $PEtOx_{50}$ in DMA (in the presence of LiBr) as eluent.

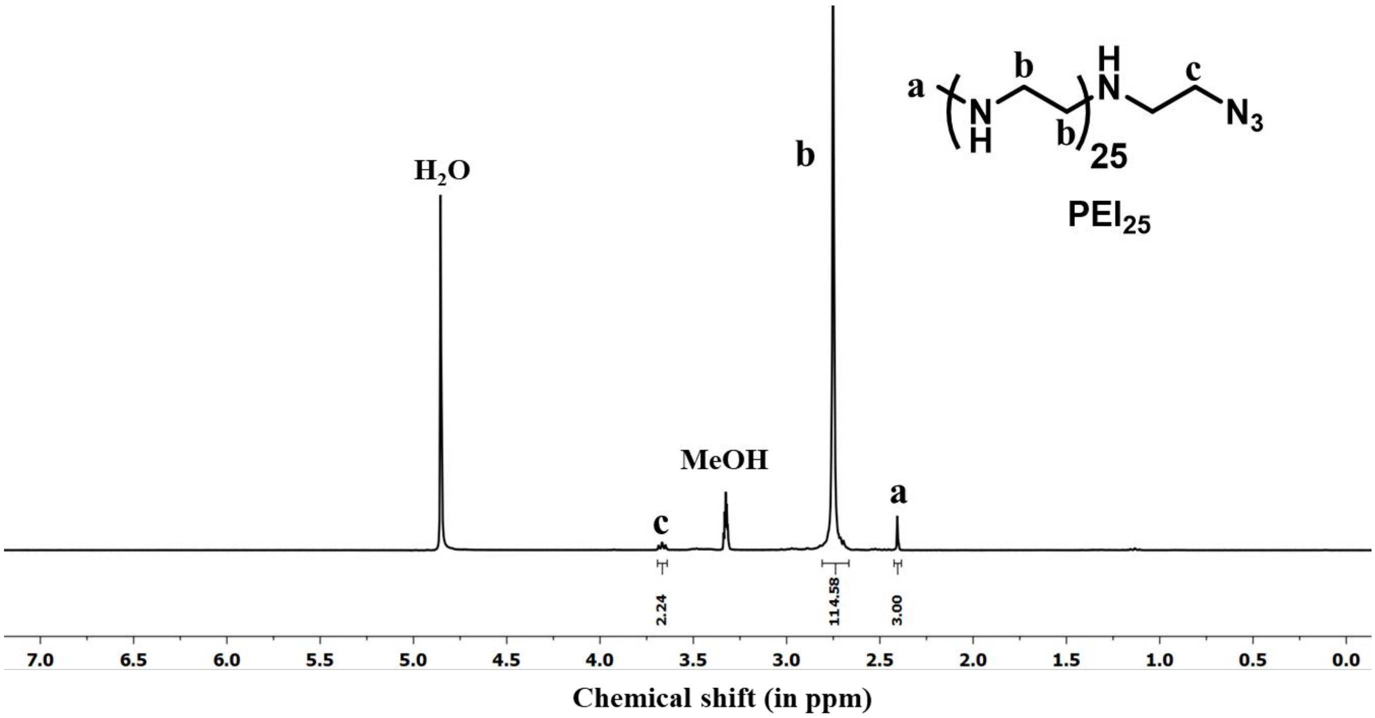

Figure S3. ^{1}H (300 MHz) NMR spectrum of PEI_{25} in CD_3OD.

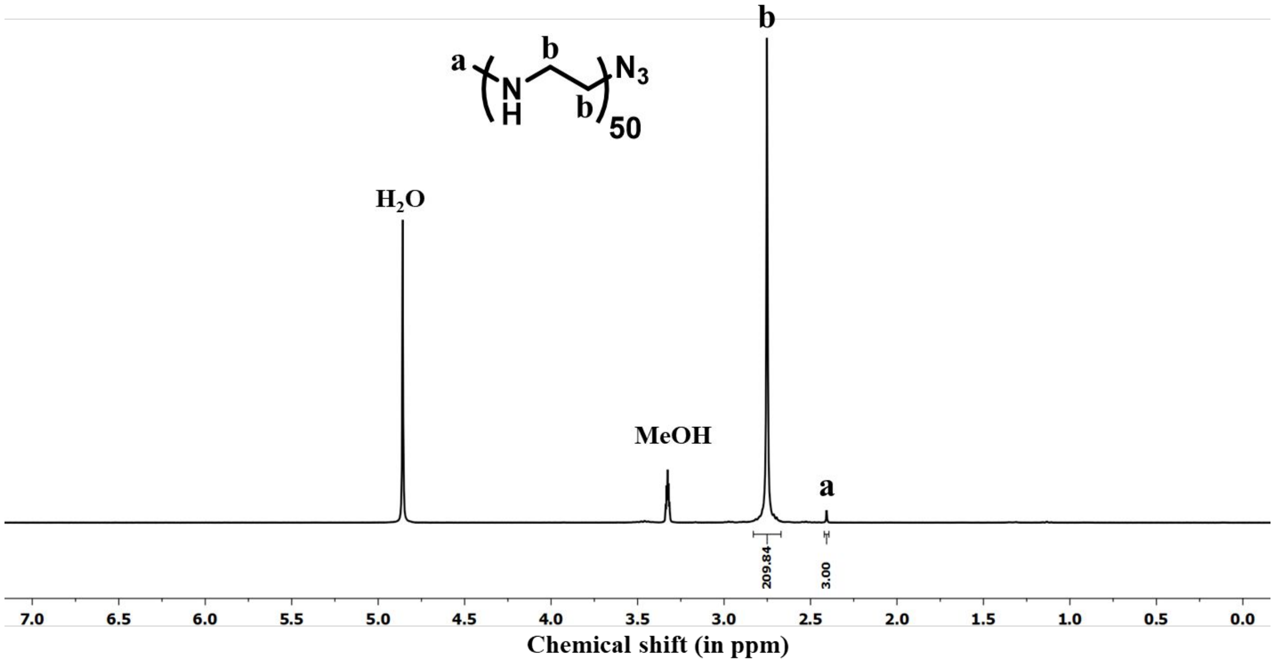

Figure S4. ^{1}H (300 MHz) NMR spectrum of PEI_{50} in CD_3OD.

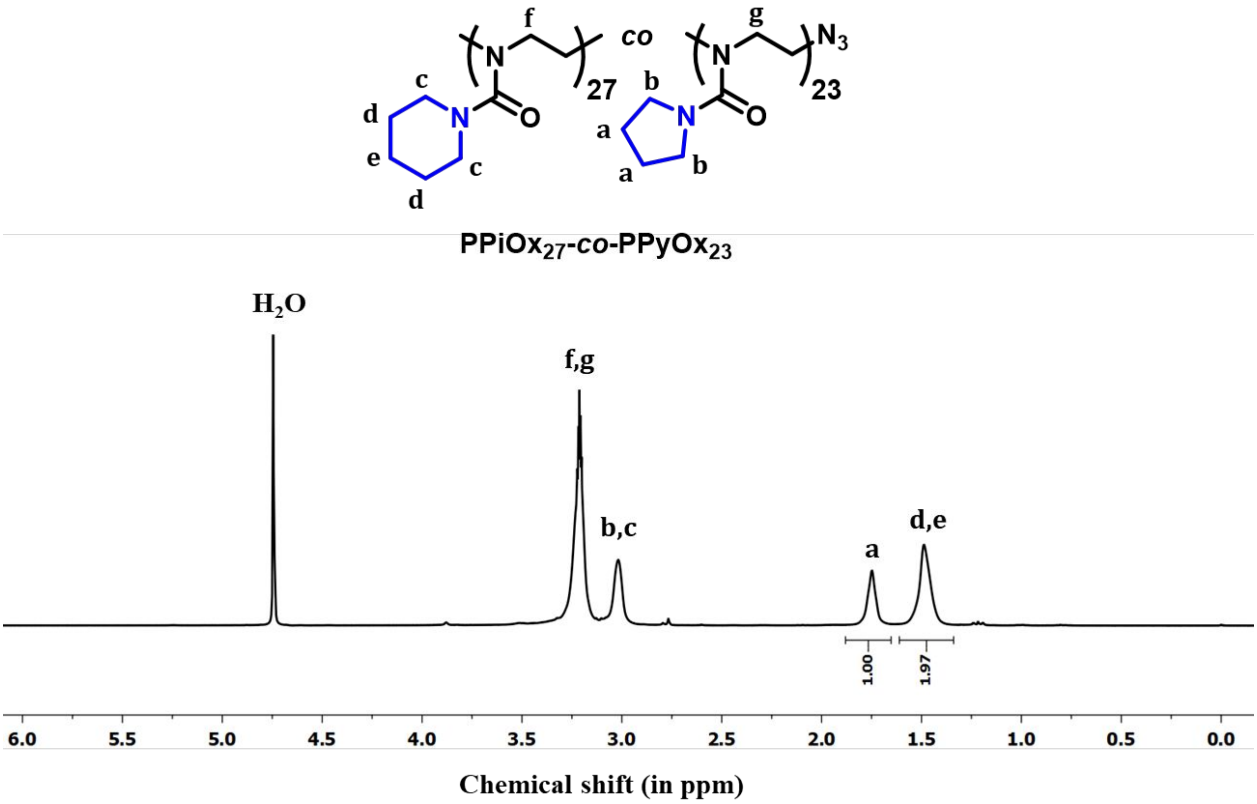

Figure S5. ^{1}H (300 MHz) NMR spectrum of PPiOx$_{27}$-*co*-PPyOx$_{23}$ in CD_3OD.

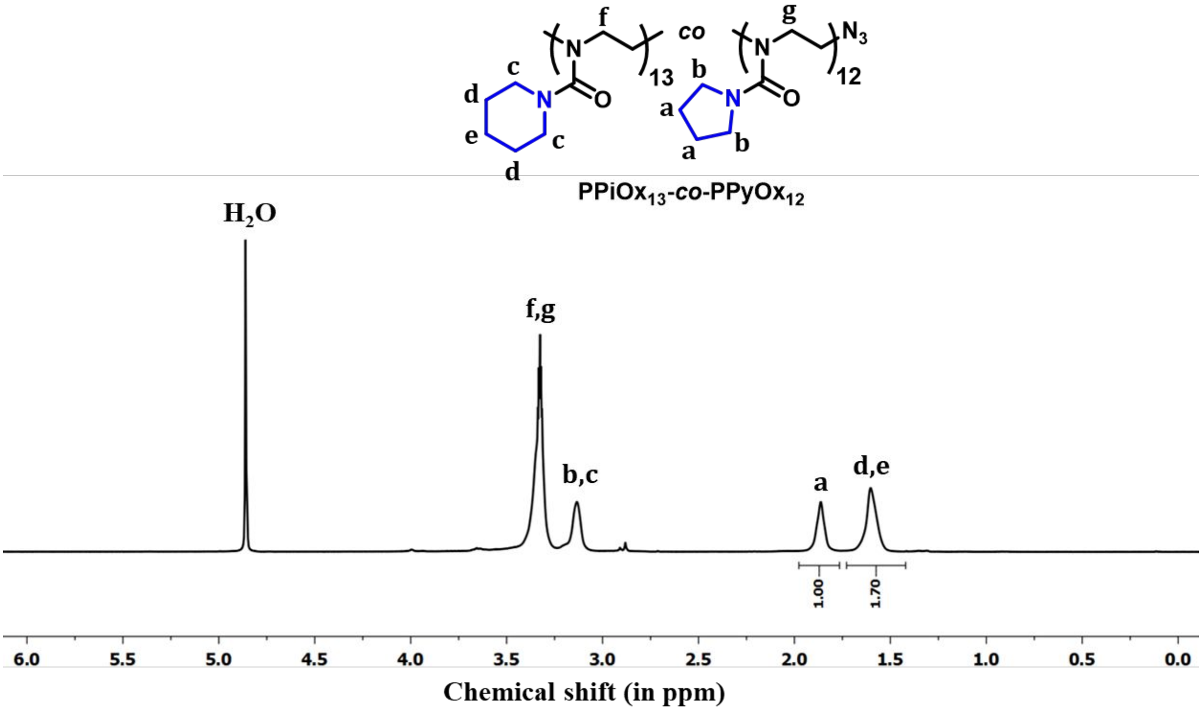

Figure S6. ^{1}H (300 MHz) NMR spectrum of PPiOx$_{13}$-*co*-PPyOx$_{23}$ in CD_3OD.

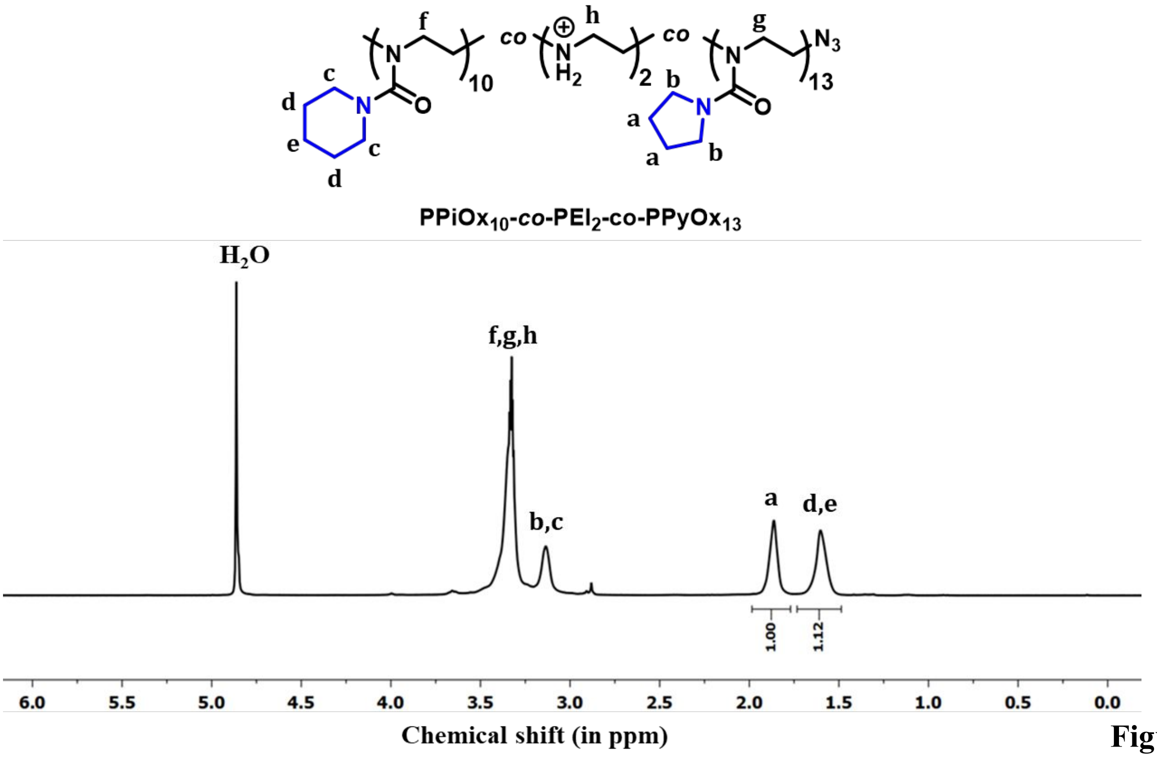

Figure S7. ^{1}H (300 MHz) NMR spectrum of PPiOx$_{10}$-*co*-PEI$_2$-*co*-PPyOx$_{13}$ in CD_3OD.

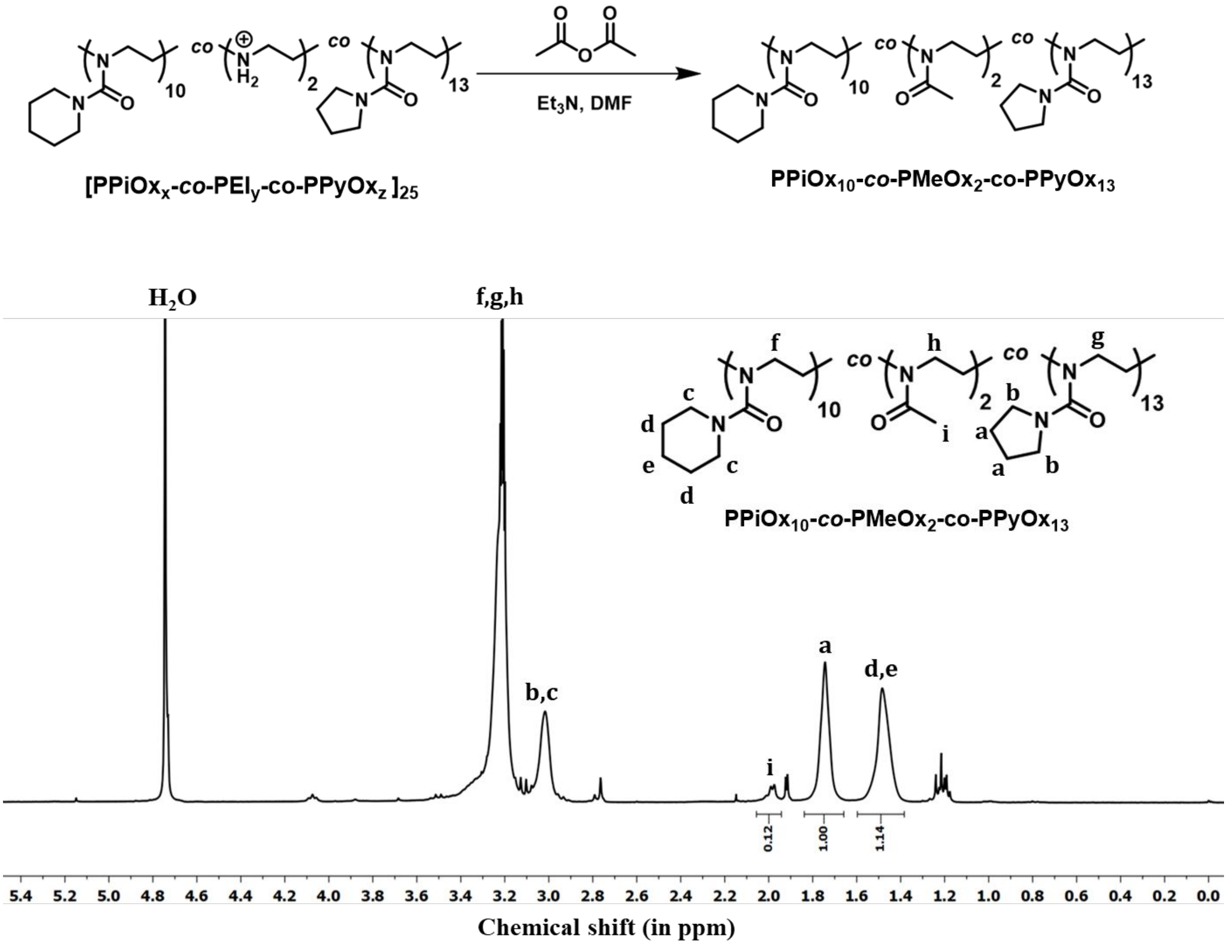

Figure S8. Representation of chemical reaction to reverse-calculate the mol% of PPiOx, PEI and PPyOx segments in the [PPiOx$_x$-*co*-PEI$_y$-*co*-PPyOx$_z$]$_{25}$ copolymer. ^{1}H (300 MHz) NMR spectrum of PPiOx$_{10}$-*co*-PMeOx$_2$-*co*-PPyOx$_{13}$ in CD_3OD.

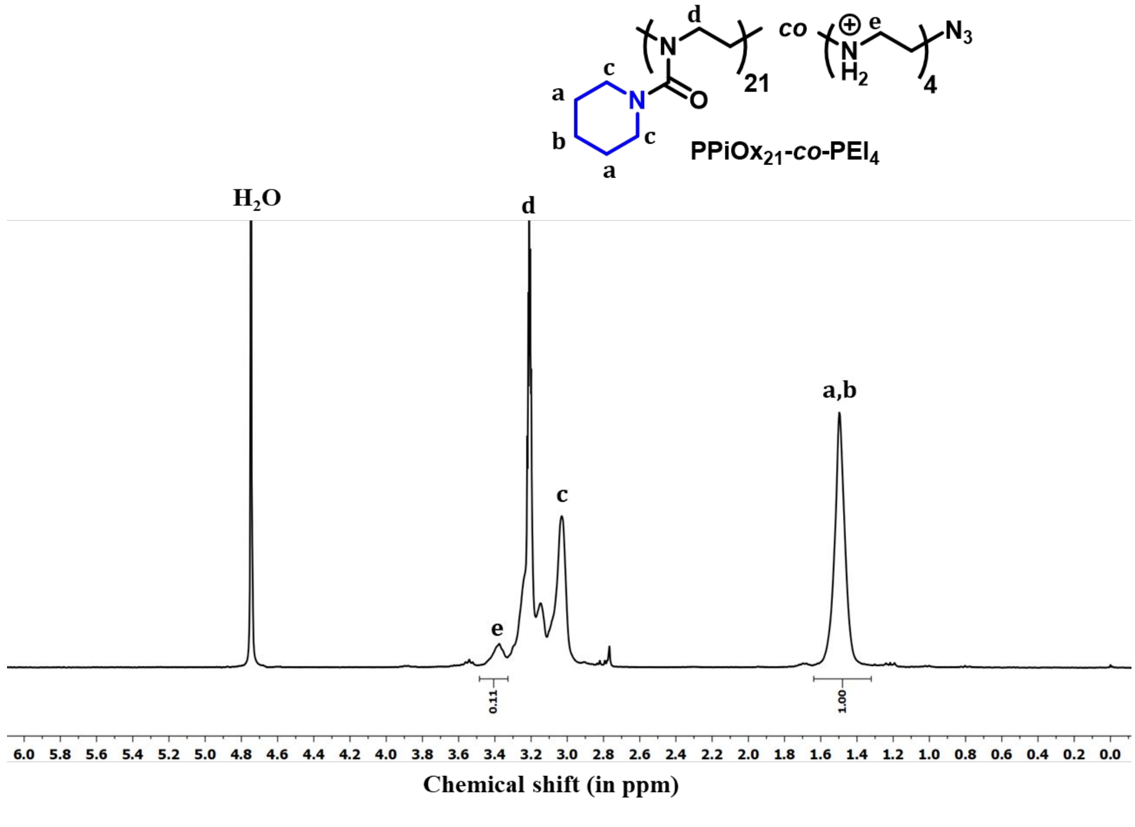

Figure S9. ^{1}H (300 MHz) NMR spectrum of $PPiOx_{21}$-*co*-PEI_4 in CD_3OD.

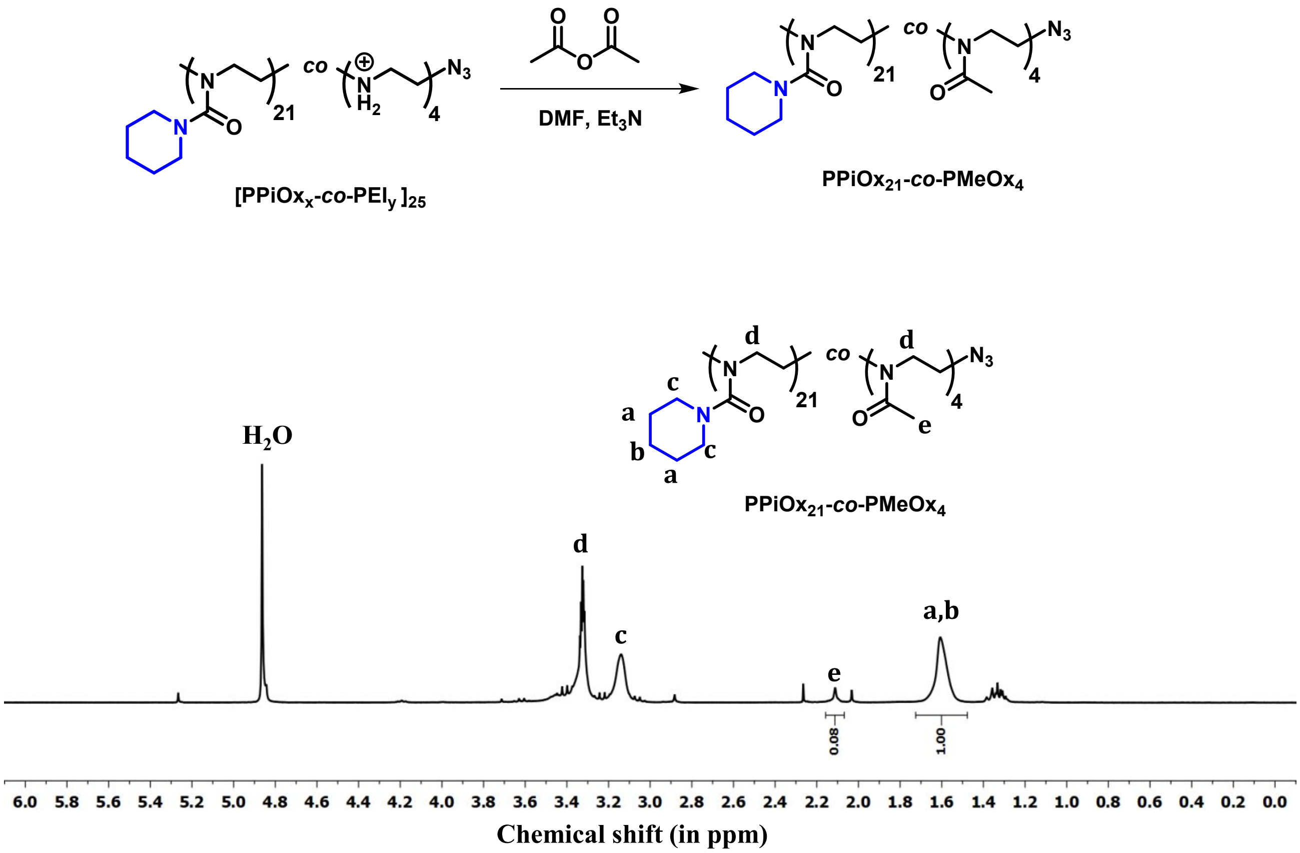

Figure S10. Representation of chemical reaction to reverse-calculate the mol% of PPiOx and PEI segments in the $[PPiOx_x$-*co*-$PEI_y]_{25}$ copolymer. ^1H (300 MHz) NMR spectrum of $PPiOx_{21}$-*co*-$PMeOx_4$ in CD_3OD.

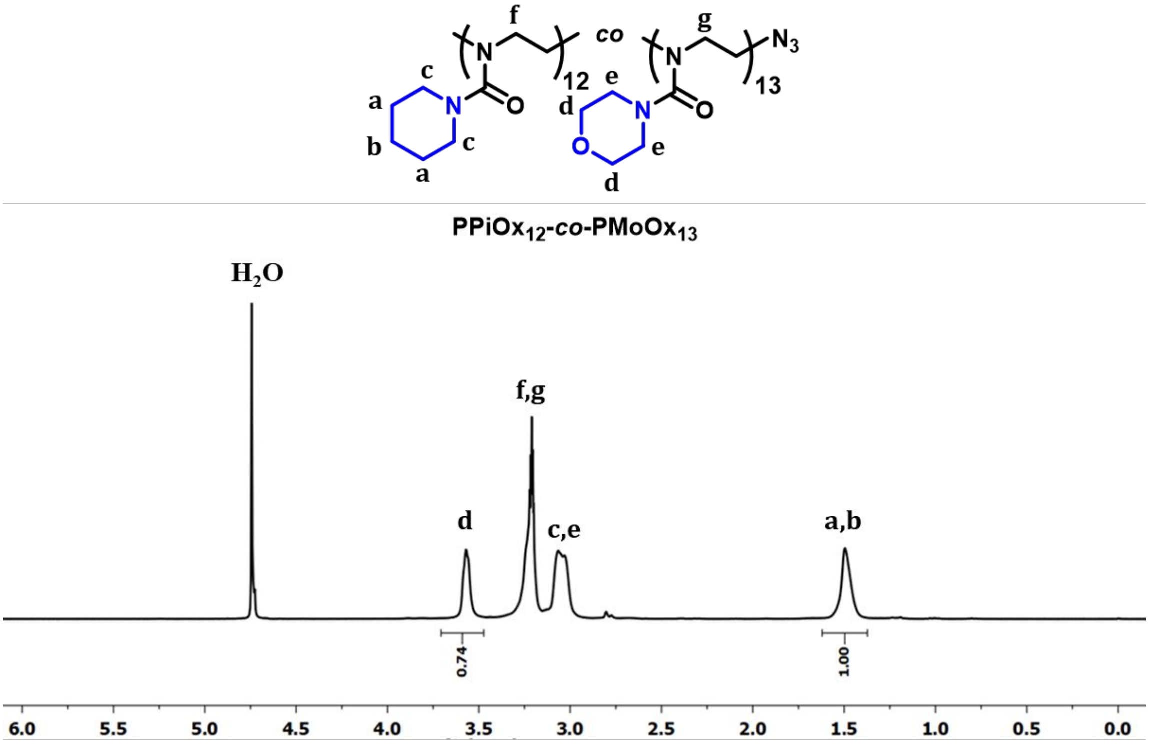

Figure S11. ^1H (300 MHz) NMR spectrum of PPiOx$_{12}$-*co*-PMoOx$_{13}$ in CD_3OD.

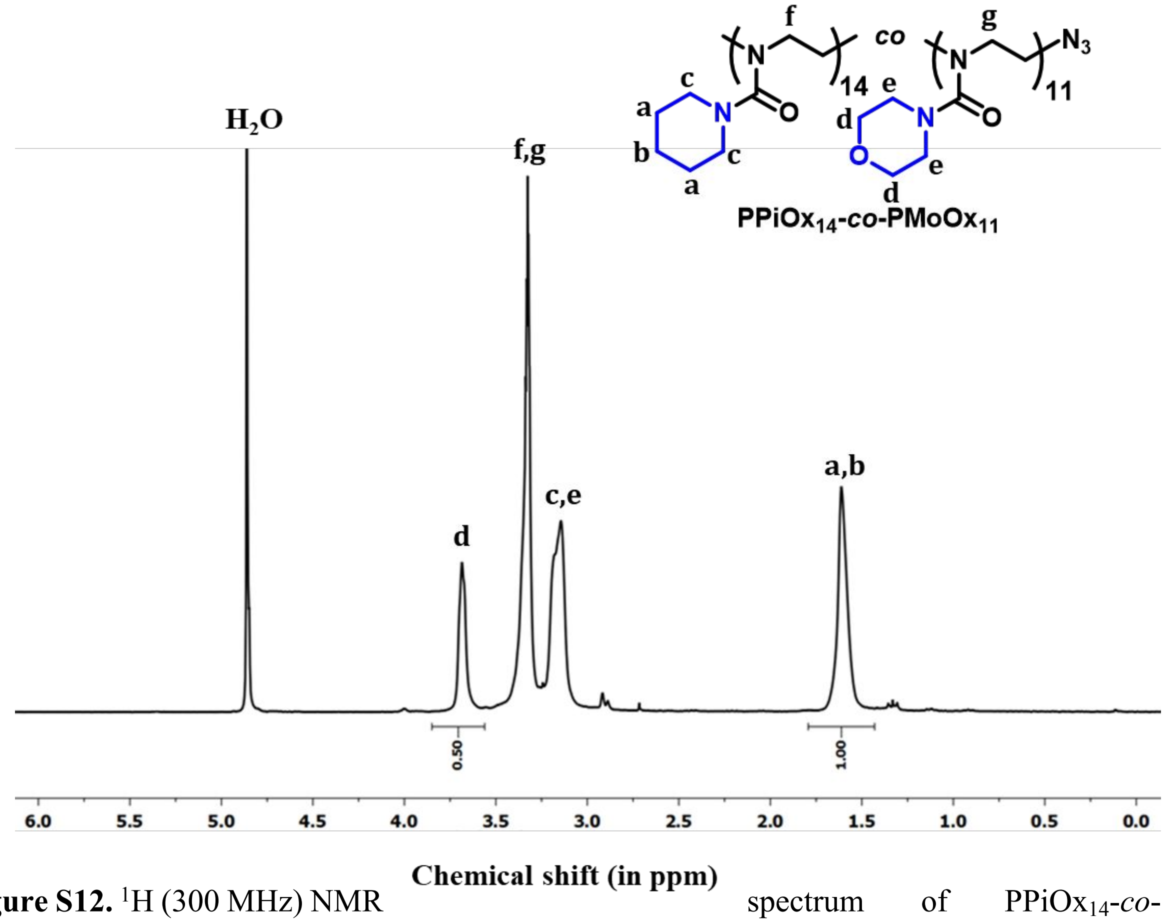

Figure S12. ^{1}H (300 MHz) NMR spectrum of PPiOx$_{14}$-*co*-PMoOx$_{11}$ in CD_3OD.

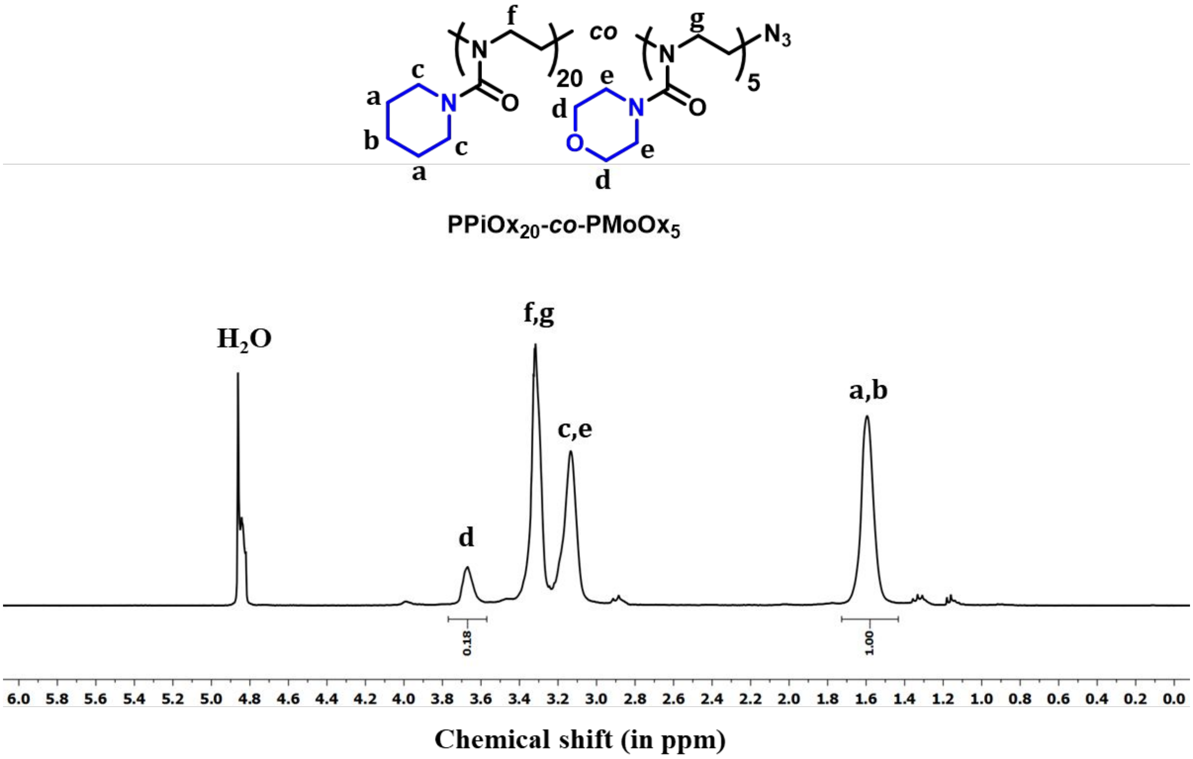

Figure S13. ^{1}H (300 MHz) NMR spectrum of $PPiOx_{20}$-*co*-$PMoOx_5$ in CD_3OD.

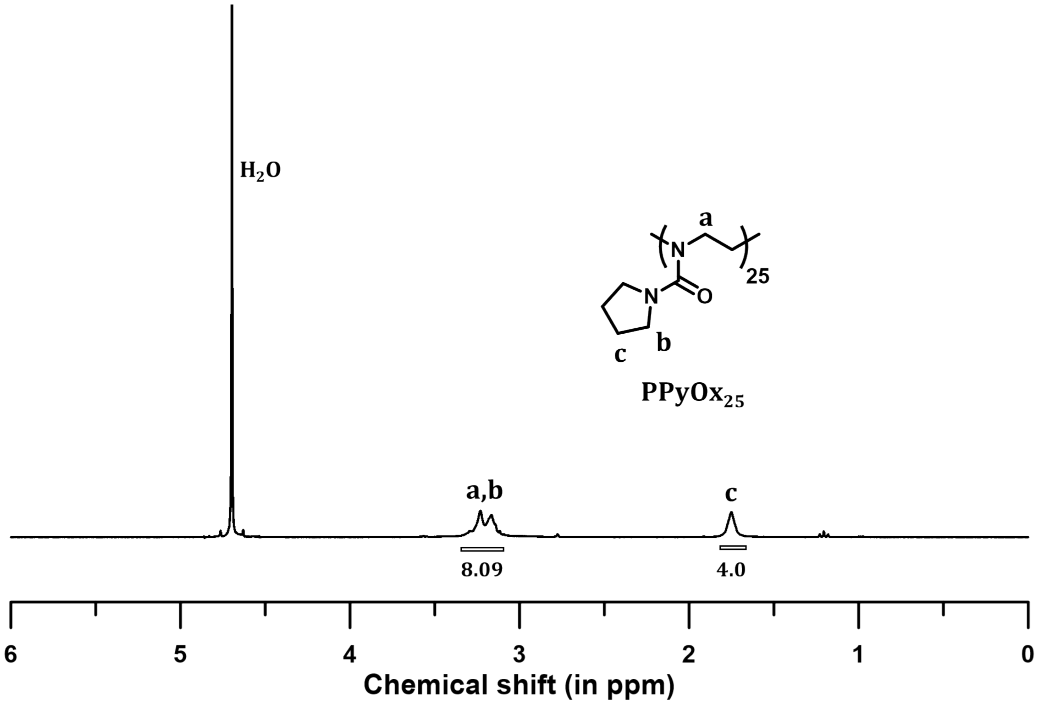

Figure S14. ^{1}H (300 MHz) NMR spectrum of PPyOx$_{25}$ in D_2O.

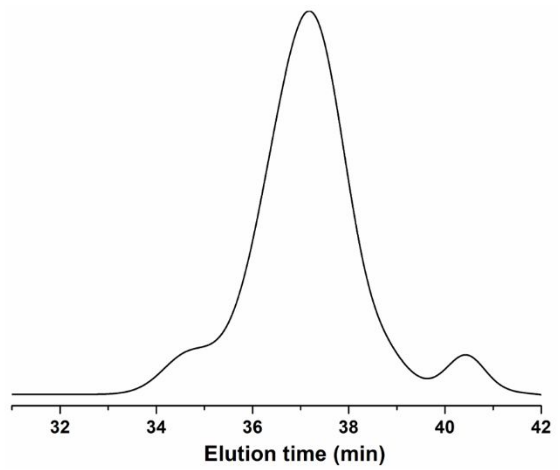

Figure S15. SEC trace of prepared $PPyOx_{25}$ in methanol-sodium acetate buffer eluent.

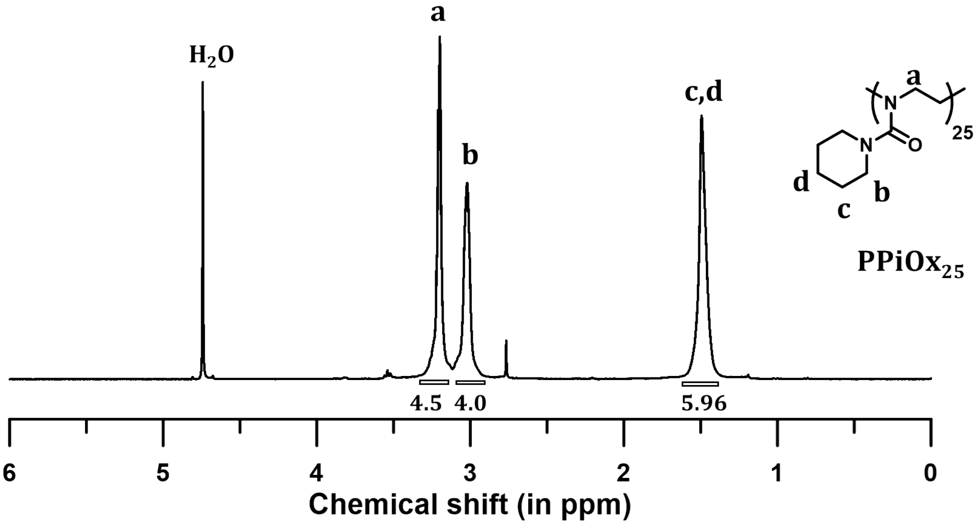

Figure S16. ^{1}H (300 MHz) NMR spectrum of $PPiOx_{25}$ in CD_3OD.

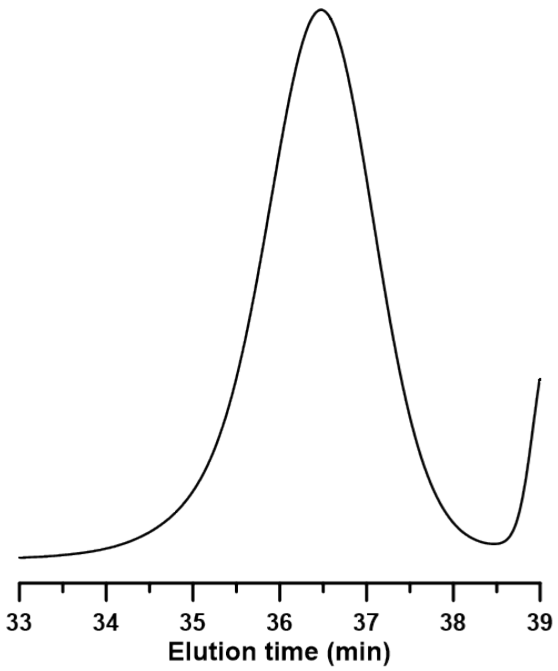

Figure S17. SEC trace of prepared $PPiOx_{25}$ in DMA (in the presence of LiBr) eluent.

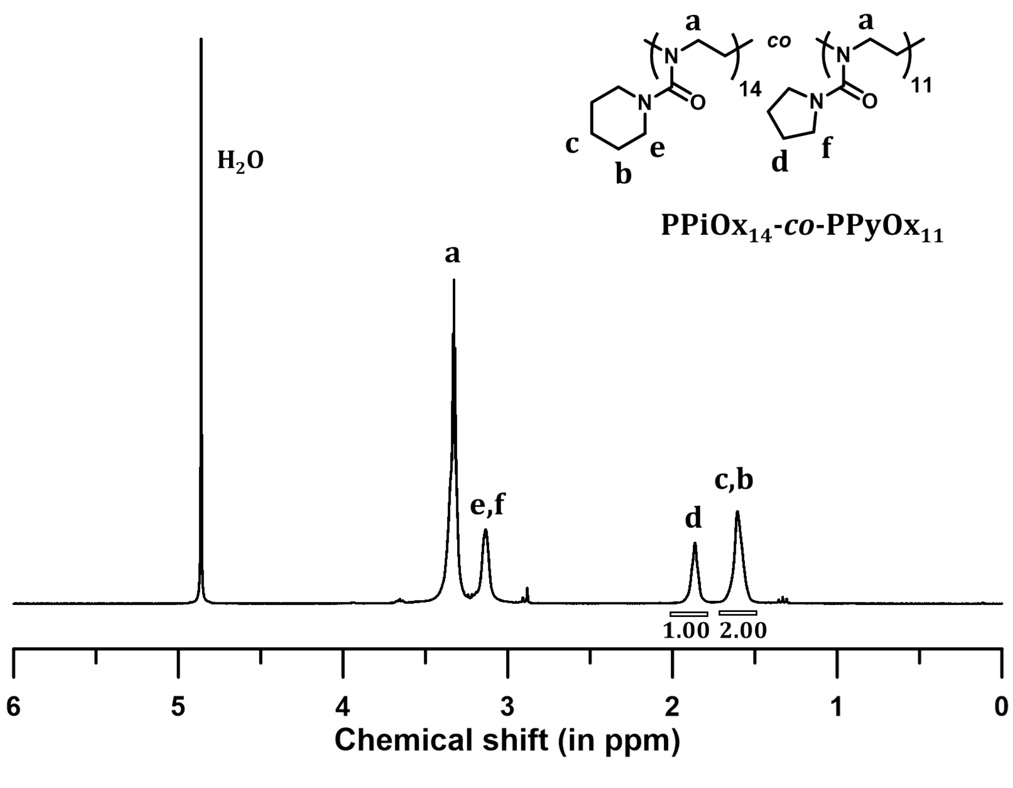

Figure S18. ^{1}H (300 MHz) NMR spectrum of PPiOx$_{14}$-*co*-PPyOx$_{11}$ copolymer in CD_3OD.

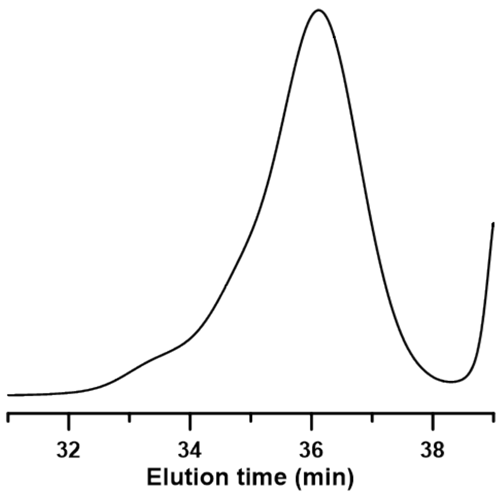

Figure S19. SEC trace of prepared PPiOx$_{14}$-*co*-PPyOx$_{11}$ copolymer in in methanol-sodium acetate buffer eluent.

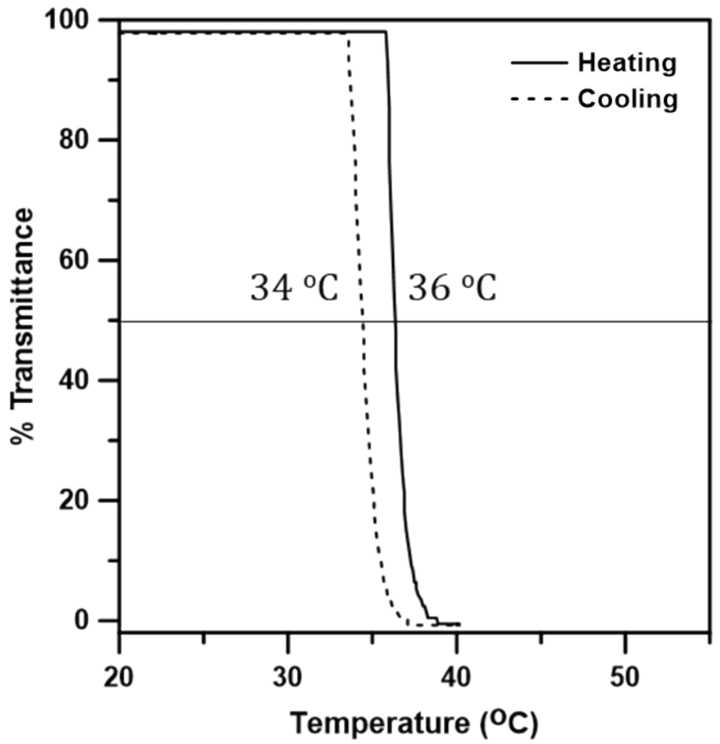

Figure S20. Turbidimetry plot of $PPiOx_{13}$-*co*-$PPyOx_{12}$ in H_2O (sample conc. 2mg/mL).

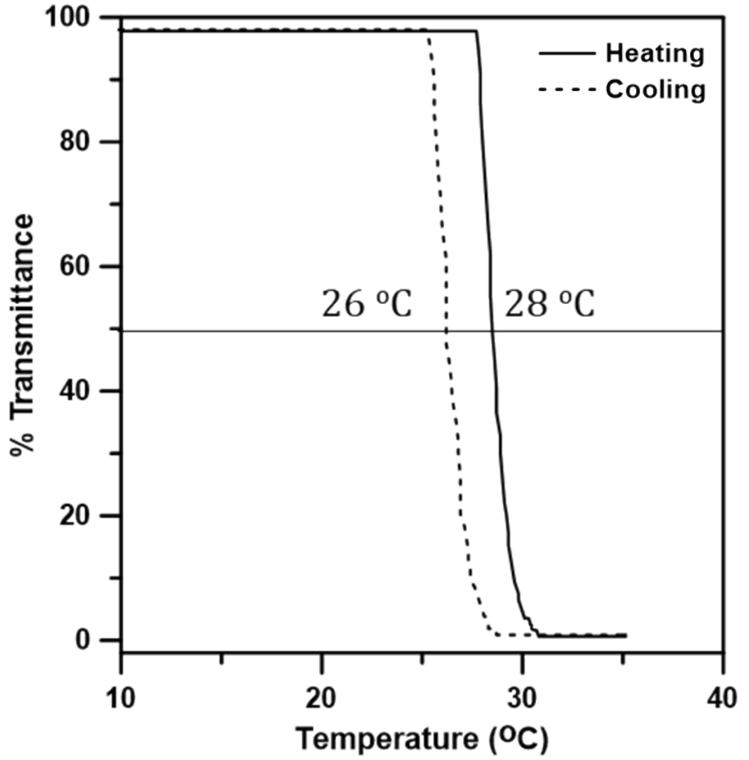

Figure S21. Turbidimetry plot of $PPiOx_{27}$-*co*-$PPyOx_{23}$ in H_2O (sample conc. 2mg/mL).

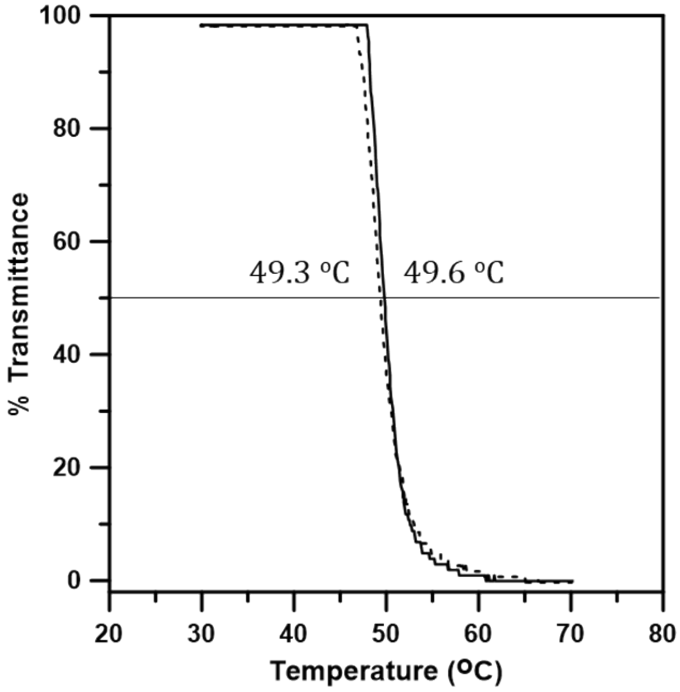

Figure S22. Turbidimetry plot of $PPiOx_{10}$-*co*-PEI_2-*co*-$PPyOx_{13}$ in H_2O (sample conc. 2mg/mL).

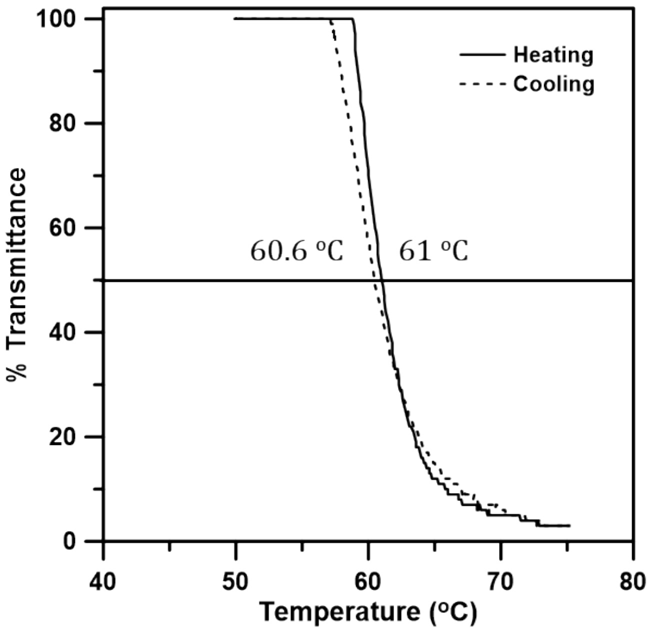

Figure S23. Turbidimetry plot of $PPiOx_{12}$-*co*-$PMoOx_{13}$ in H_2O (sample conc. 2mg/mL).

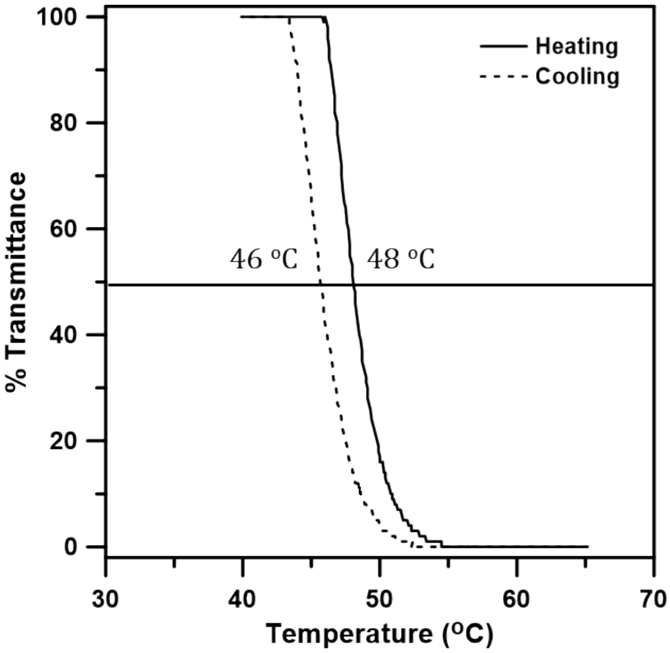

Figure S24. Turbidimetry plot of $PPiOx_{14}$-*co*-$PMoOx_{11}$ in H_2O (sample conc. 2mg/mL).

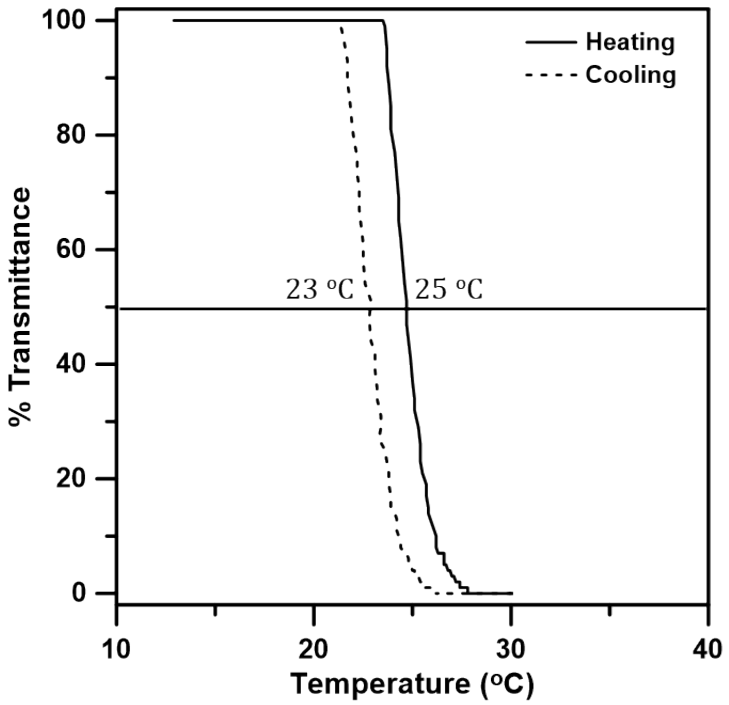

Figure S25. Turbidimetry plot of $PPiOx_{20}$-*co*-$PMoOx_5$ in H_2O (sample conc. 2mg/mL).

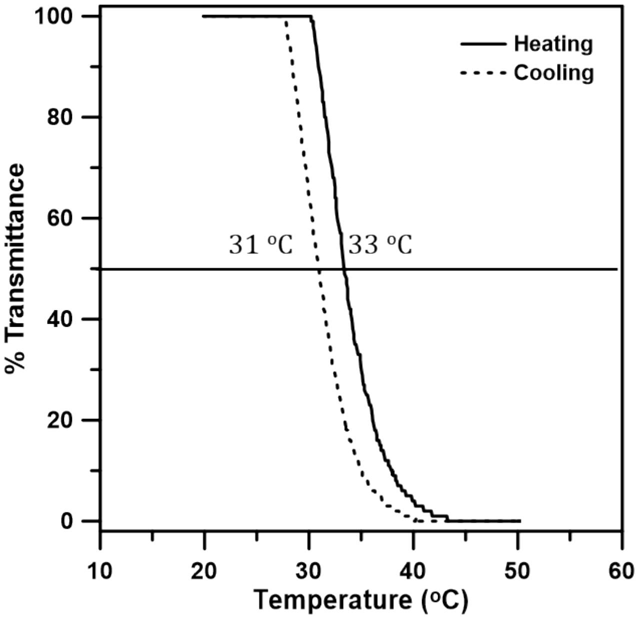

Figure S26. Turbidimetry plot of $PPiOx_{21}$-*co*-PEI_4 in H_2O (sample conc. 2mg/mL).

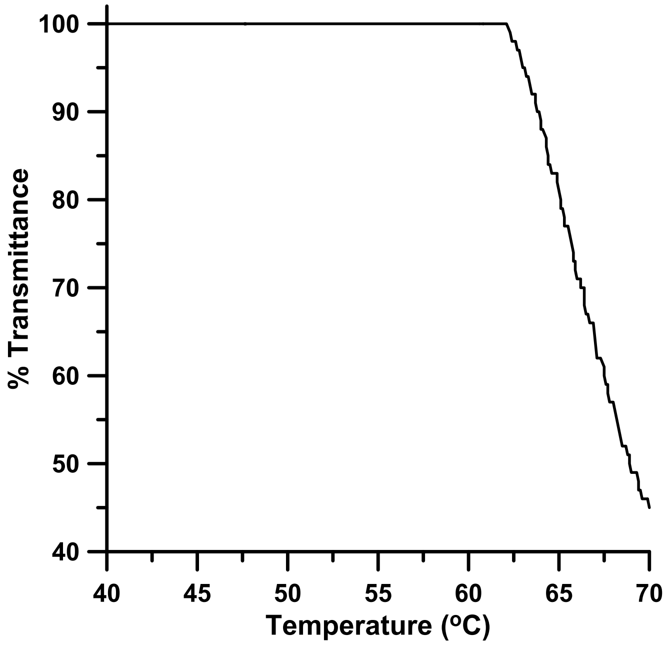

Figure S27. Turbidimetry plot of $PPyOx_{25}$ homopolymer in H_2O (sample conc. 2mg/mL).

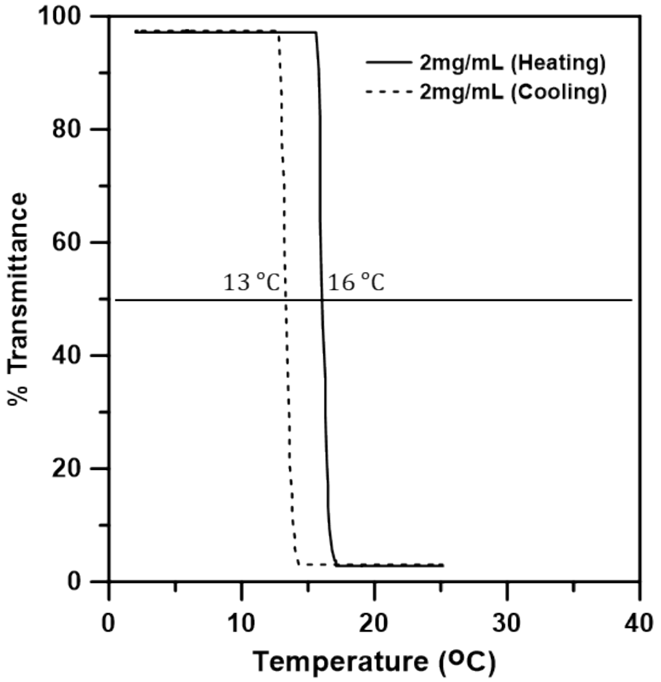

Figure S28. Turbidimetry plot of $PPiOx_{25}$ homopolymer in H_2O (sample conc. 2mg/mL).

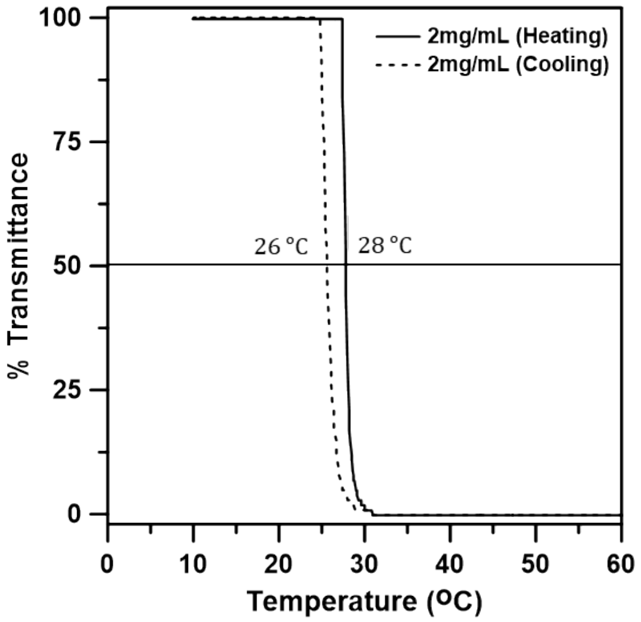

Figure S29. Turbidimetry plot of $PPiOx_{14}$-*co*-$PPyOx_{11}$ homopolymer in H_2O (sample conc. 2mg/mL).

References

1. Wiesbrock, F.; Hoogenboom, R.; Leenen, M. A. M.; Meier, M. A. R.; Schubert, U. S., Investigation of the Living Cationic Ring-Opening Polymerization of 2-Methyl-, 2-Ethyl-, 2-Nonyl-, and 2-Phenyl-2-oxazoline in a Single-Mode Microwave Reactor. *Macromolecules* **2005**, 38 (12), 5025-5034.
2. Lambermont-Thijs,H. M. L. van der Woerdt, F. S.; Baumgaertel, A.; Bonami, L.; Du Prez, F. E.; Schubert, U. S.; Hoogenboom, R., Linear Poly(ethylene imine)s by Acidic Hydrolysis of Poly(2-oxazoline)s: Kinetic Screening, Thermal Properties, and Temperature-Induced Solubility Transitions. *Macromolecules* **2010**, 43 (2), 927-933
3. Kelland, Malcolm A., Somdeb Jana, Janronel Pomicpic, Ondrej Sedlacek, and Richard Hoogenboom. "Kinetic Hydrate Inhibition from Thermoresponsive Poly (2-amino-2-oxazoline) s: Size and Shape of the Hydrophobic Groups Are Critical for Performance." Energy & Fuels 38, no. 5 (2024): 3784-3791.
